# Using the Jurkat reporter T cell line for evaluating the functionality of novel chimeric antigen receptors

**DOI:** 10.3389/fmmed.2023.1070384

**Published:** 2023-02-22

**Authors:** Farhana Jahan, Jan Koski, Diana Schenkwein, Seppo Ylä-Herttuala, Helka Göös, Sini Huuskonen, Markku Varjosalo, Pilvi Maliniemi, Judith Leitner, Peter Steinberger, Hans-Jörg Bühring, Kim Vettenranta, Matti Korhonen

**Affiliations:** ^1^ R&D, Finnish Red Cross Blood Service, Helsinki, Finland; ^2^ A.I Virtanen Institute for Molecular Sciences, University of Eastern Finland, Kuopio, Finland; ^3^ Gene Therapy Unit, Kuopio University Hospital, Kuopio, Finland; ^4^ Institute of Biotechnology, HiLIFE, University of Helsinki, Helsinki, Finland; ^5^ Division of Immune Receptors and T Cell Activation, Institute of Immunology, Center for Pathophysiology, Infectiology and Immunology, Medical University of Vienna, Vienna, Austria; ^6^ Department of Internal Medicine II, University Clinic of Tübingen, Tübingen, Germany; ^7^ University of Helsinki and the Children’s Hospital, University of Helsinki, Helsinki, Finland

**Keywords:** FiCAR, CAR (chimeric antigen receptor) T cells, cancer, phosphoprotein, cell signaling

## Abstract

**Background:** T cells that are genetically modified with chimeric antigen receptor (CAR) hold promise for immunotherapy of cancer. Currently, there are intense efforts to improve the safety and efficacy of CAR T cell therapies against liquid and solid tumors. Earlier we designed a novel CAR backbone (FiCAR) where the spacer is derived from immunoglobulin (Ig) -like domains of the signal-regulatory protein alpha (SIRPα). However, the analysis of novel CAR using primary T cells is slow and laborious.

**Methods:** To explore the versatility of the CAR backbone, we designed a set of variant FiCARs with different spacer lengths and targeting antigens. To expedite the analysis of the novel CARs, we transduced the FiCAR genes using lentiviruses into Jurkat reporter T cells carrying fluorescent reporter genes. The expression of fluorescent markers in response to FiCAR engagement with targets was analyzed by flow cytometry, and cytotoxicity was evaluated using killing assays. Furthermore, the killing mechanisms that are employed by FiCAR-equipped Jurkat T cells were investigated by flow cytometry, and the intracellular pathways involved in signaling by FiCAR were analyzed by phosphoproteomic analysis using mass spectrometry.

**Results:** Seven different CARs were designed and transduced into Jurkat reporter cells. We show that the SIRPα derived FiCARs can be detected by flow cytometry using the SE12B6A4 antibody recognizing SIRPα. Furthermore, FiCAR engagement leads to robust activation of NFκβ and NFAT signaling, as demonstrated by the expression of the fluorescent reporter genes. Interestingly, the Jurkat reporter system also revealed tonic signaling by a HER-2 targeting FiCAR. FiCAR-equipped Jurkat T cells were cytotoxic in cocultures with target cells and target cell engagement lead to an upregulation of CD107a on the Jurkat reporter T cell surface. Phosphoproteomic analyses confirmed signal transduction *via* the intracellular CD28/CD3ζ sequences upon the interaction of the FiCAR1 with its antigen. In addition, downstream signaling of CD3ζ/ZAP70- SLP-76-PLCγ, PI3K–AKT–NFκB pathways and activation of NFAT and AP-1 were observed.

**Conclusion:** We conclude that the FiCAR backbone can be shortened and lengthened at will by engineering it with one to three SIRPα derived Ig-like domains, and the FiCARs are functional when equipped with different single chain variable fragment target binding domains. The Jurkat reporter system expedites the analysis of novel CARs as to their expression, signaling function, evaluation of tonic signaling issues and cytotoxic activity.

## 1 Introduction

Genetically modified T cells are an emerging immunotherapy approach: the patient’s own immune cells are collected, genetically modified, and applied to treat their cancer. Several approaches have been used for cell modification, and presently chimeric antigen receptor (CAR) T cell therapy is furthest along in clinical development. CAR T cells have shown significant efficacy in the treatment of refractory B-lineage acute lymphoblastic leukemia (ALL) and diffuse large B cell lymphoma (DLBCL) (Lamure et al., 2021) both in children and adults as well as multiple myeloma (Salter et al., 2018; van de Donk et al., 2021). However, many challenges remain in improving the safety and efficacy of CAR T cell therapy for liquid malignancies and extending it to the treatment of solid tumors. In our earlier work, we have identified cell culture components that allow the generation of CAR T cells of early memory T cell phenotype (Kaartinen et al., 2017), and screened small molecule drugs that can be used to enhance CAR T cell activity (Dufva et al., 2020).

The generic CAR comprises an extracellular target binding domain, a hinge and spacer region, a transmembrane domain (TM), and an intracellular signaling domain (Pan et al., 2022). In our earlier work, we used a version of CAR that contains a spacer derived from the IgG1 crystallizable fragment (Fc) that interacts with myeloid cells carrying Fc receptors (FcR) (Dufva et al., 2020). This interaction leads to CAR T cell and macrophage activation, macrophage killing and activation-induced cell death (AICD) causing inhibition of CAR T cell activity *in vivo* (Hombach et al., 2010; Almåsbak et al., 2015; Hudecek et al., 2015; Koski et al., 2022). To circumvent this problem, we designed a novel CAR where the spacer region is derived from Ig-like C1-type 1 and -2 domains from signal-regulatory protein alpha (SIRPα) and showed that these FiCARs evade unwanted interactions with monocytes (Koski et al., 2022). SIRPα is a membrane glycoprotein that recognizes CD47, an anti-phagocytic signal which discriminates living cells from dying cells (Barclay and Brown, 2006). SIRPα interacts with CD47 *via* its N-terminal V-type Ig-like domain (Hatherley et al., 2009). In addition, the ability to modify the length and the target specificity of FiCARs would be advantageous. To this end, we made five different versions of the CD19 targeting FiCAR and additionally designed CARs where we replaced the CD19 targeting scFv with ones targeting HER-2 (a breast cancer antigen) and GD2 (neuroblastoma antigen).

To improve the potency of the treatment, systematic characterization and assessment of CARs are required. Researchers commonly use primary T cells from random healthy donors for CAR T cell production, which requires isolation, genetic modifications, and expansion of cells before analysis can be made. Therefore, the whole process is laborious, time-consuming and expensive before we can determine whether a particular CAR is functional. Also, donor-to-donor variation is seen, affecting the reproducibility of CAR production and evaluation (Golubovskaya and Wu, 2016; Sommermeyer et al., 2016).

The human Jurkat T cell line has been an important research tool for interrogating the function of the T cell receptor (TCR)s (Aarnoudse et al., 2002; Anmole et al., 2015; Rosskopf et al., 2018; Müller et al., 2020). Jurkat cells have also been used for the screening of novel CARs for various cancers (Darowski et al., 2019; Bloemberg et al., 2020; Hassani et al., 2020; Kang et al., 2021). The Jurkat cell line JE6.1 has been engineered with genetic constructs that result in the expression of enhanced cyanine fluorescence protein (eCFP), enhanced green fluorescence protein (eGFP) and m-cherry upon the activation in the cells of the transcription factors, nuclear factor kappa-light-chain-enhancer of activated B cells (NFκβ), nuclear factor of activated T cells (NFAT) and activation protein 1 (AP-1), respectively (Jutz et al., 2016). This Jurkat reporter line can be used to evaluate T cell responses to cancer or virus-specific antigens and can also be harnessed to evaluate the function of signaling receptors (Rydzek et al., 2019; Müller et al., 2020). In this study, we have used the Jurkat reporter T cell line as a tool for the development and early assessment of the function of novel CARs.

We show that the SIRPα derived FiCARs are expressed and functional in Jurkat reporter cells. Furthermore, we also found that FiCAR-equipped Jurkat T cells are cytotoxic in cocultures with target cells. These findings were corroborated by expressing the FiCARs also in primary T cells. Importantly, the Jurkat reporter cells reveal antigen-independent activation by a HER-2 targeting FiCAR which is crucial information for further CAR development. Additionally, phosphoproteomic analyses by mass spectrometry (MS) revealed that the FiCAR signaling pathway in Jurkat reporter T cells comprises the PI3K–AKT–mTOR, NFAT, NF-κβ, and MAPK pathways.

## 2 Materials and methods

### 2.1 Cell lines

The Jurkat reporter T cell and the Nalm-6-luciferase positive (luc+) cell lines were generous gifts from Peter Steinberger and Satu Mustjoki, respectively. The generation of Jurkat reporter cells and the luc + cells has been described previously (Jutz et al., 2016; Dufva et al., 2020). The Nalm-6 cell line (CD19^+^ B lineage, acute lymphoblastic leukemia, ALL) was a gift from Olli Lohi (University of Tampere, Finland). Nalm-6 and Jurkat T cells were cultured in RPMI-1640 medium (Life Technologies) supplemented with 10% fetal bovine serum (FBS) (Life Technologies), 2 mM L-glutamine, and 100 U/mL penicillin-streptomycin (Life Technologies). The NIH/3T3 (mouse embryonic fibroblast) cell line was a gift from Heikki Joensuu (University of Helsinki, Finland) and this cell line was maintained with DMEM (Dulbecco’s modified Eagle’s medium, cat#. 302002. The American Type Culture Collection (ATCC)), 10% bovine calf serum (cat#. 12138C SAFC Biosciences, USA), and 100 IU/mL penicillin-streptomycin (PS) (Life Technologies). SKBR3 (ATCC #HTB-30) breast cancer target cells were cultured in McCoy’s 5a medium (ATCC, Catalog No. 30-2007) supplemented with 10% FBS and 100IU/mL of PS. The cell lines were regularly tested for *mycoplasma* contamination.

### 2.2 CAR constructs and transduction

The original structures of FiCAR1 and FiCAR2 were described earlier (Koski et al., 2022). Briefly, the CD19-targeting scFv is from the FMC63 antibody (Genbank: immunoglobulin light chain (VL), variable region; CAA74660.1 and immunoglobulin heavy chain (VH), variable region; CAA74659.1). The hinge region from the IgG1-CH1 domain linked the scFv with the spacer. The SIRPα Ig-like C1-type 1 and C1-type 2 were used as novel spacers for the FiCAR1 and FiCAR2 (Koski et al., 2022). The SIRPα sequences were obtained from the UniProt database (P78324-1). TM and intracellular domains were from the T cell-specific surface glycoprotein CD28 (UniProt P10747-1) and the intracellular T lymphocyte activation domain of the T cell receptor (TCR, CD3)-zeta (ζ)-chain; (UniProt P20963-3). Different versions of the SIRPα spacer were designed either by removing the C1-type 1 domain FiCAR1-short S)) or adding an extra C1-type 1 domain FiCAR1-long L). FiCAR2 contains a juxtamembrane cysteine from CD28. We added a second C1-type 2 domain onto the FiCAR2 structure to create the FiCAR2 extra long (XL) structure.

To change the targeting moieties in the FiCAR1 spacer-backbone, the FMC63 scFv was replaced with scFv’s from the humAb4D5-5 antibody (Carter et al., 1992) targeting HER-2 (breast cancer) and from the ch14.18 antibody targeting GD2 (neuroblastoma) (Gillies et al., 1989).

All the primary sequences were reverse translated utilizing human codons employing estimated probabilities based on frequency distribution (Quax et al., 2015) and assembled *in silico* using the SnapGene^®^ -software (from GSL Biotech; available at snapgene.com). Assembled sequences were synthesized at Genewiz (Azenta Inc. Massachusetts, USA)) and plasmids packed into Lentiviral vectors (LV) (Schenkwein and Turkki, 2010) in the National Virus Vector Laboratory at the A.I. Virtanen Institute for Molecular Sciences (University of Eastern Finland, Finland).

As a comparator control an IgG-based CAR (FMC63 scFv, IgG1-CH2-CH3 spacer, CD28 TM, followed by CD28 and CD3ζ-signaling intracellular domain, a generous gift from Dr. Gianpietro Dotti, (University of North Carolina, USA) was used and an empty LV (mock) served as a negative control.

The Jurkat reporter cells were thawed and cultured for 3 days before transduction. 1 × 10^6^ Jurkat reporter cells were transduced with lentiviruses carrying the CD19, HER-2 and GD2 targeting FiCARs with a multiplicity of infection (MOI) of 5.24 h post-transduction, the medium was removed, the cells were washed once with phosphate-buffered saline (PBS) and sub-cultured at a density of 0.5 × 10^6^/mL in fresh cell culture medium. To obtain the murine CD19^+^ target cell line, we transduced NIH/3T3 cells with truncated CD19 lacking the intracellular signaling domain (UniProt P15391-1, AA 19-316)). The sequence was codon optimized (EMBOSS Backtranseq) using *Homosapiens* preferred codons. Cells were transduced at MOI 5, and 8 μg/mL polybrene (Sigma-Aldrich# TR-1003-G) was used to increase transduction efficiency. The medium was removed 32 h post-infection and the cells were washed with PBS. The cells were detached with TrypLE solution (A12859-01, Gibco) and subcultured at a density of 2–3X10^3^ cells/cm^2^ twice weekly. CD19-expressing 3T3 cells were enriched using anti-CD19 microbead selection according to the manufacturer’s instructions (Miltenyi Biotech, Germany).

### 2.3 Production of CAR T cells from primary T cells

CAR T cells were manufactured from two healthy donors from CD4 and CD8 positive T cells according to Miltenyi’s protocol. Briefly, buffy coats from voluntary donors, not required for the treatment of patients were obtained from the FRCBS under an institutional permit (FRCBS 178/39/2019). Peripheral blood mononuclear cells (PBMC) were separated from buffy coats using Ficoll-Paque Premium (GE Healthcare, Chicago, USA) density gradient separation, and T cells were isolated from PBMCs using CD4^+^ and CD8^+^ microbeads (CD4 microbeads, human 130-045-101, CD8 microbeads, human 130-045-201 Miltenyi) and activated with T Cell TransAct™ (Miltenyi, 130-111-160) (day 1) in TexMACS medium (Miltenyi, 130-097-196) supplemented with 12.5 ng/mL of IL-7 (Miltenyi, 130-095-361) and IL-15 (Miltenyi, 130-095-762). The following day (day 2) T cells were transduced with the different versions of FiCAR CD19 constructs using LVs and incubated for 48 h (day 4). An empty lentiviral vector (mock) was used as a negative control for the CD19 CAR constructs. On day 4, the cell culture supernatant consisting of T cell TransAct and LVs was removed and CAR T cells were expanded in TexMACS medium supplemented with IL-7 and IL-15. CAR T cells were subcultured every 2 days. On day 10, CAR expression was analyzed with flow cytometry and functional assays were performed.

### 2.4 Flow cytometry and imaging flow cytometry

For the detection of CD19 CAR expression a biotinylated CD19 CAR detection reagent kit, human, (130-115-965, Miltenyi) and as a biotin recognition reagent anti-biotin-APC (130-110-952, Miltenyi) were used. The SIRPα antibody (clone SE12B6A4) (Seiffert et al., 2001) was conjugated with Cy5 fluorophore using the *Lynx* rapid plus Cy5 antibody conjugation kit™ (Bio-Rad) according to the manufacturer’s protocol. For CD19 FiCAR detection 0.2–0.5 × 10^6^ cells were stained with CD19 CAR detection reagent for 20 min in the dark and then washed twice with FACS buffer (0.5% albunorm (200 g/L Octapharma) and 2 mM EDTA in PBS) at 300 g for 5 min and incubated with anti-biotin-APC for 30 min at +4°C in the dark. For FiCAR detection with the SIRP-α ab, cells were incubated with SIRPα-Cy5 ab for 30min at +4°C, then washed twice with FACS buffer and fixed with 1% paraformaldehyde for 30 min at +4°C. Data was acquired with BD FACSAria IIU (BD Biosciences) and DxFLEX (Beckman Coulter) flow cytometers using FACSDiva™ (v8.0.1, BD Biosciences) and CytExpert (Beckman Coulter) softwares respectively, and analyzed with FlowJo^®^ software (v10, BD Biosciences).

Images were taken with Amnis^®^ ImageStream®X Mark II 12-channel imaging flow cytometer with the INSPIRE^®^ software (Luminex Corporation). Excitation lasers 405 (off), 488 (15 mW), 642 (120 mW) and 785 (off) were applied for the excitation of fluorochromes and laser Channels (Ch) 01 (bright field, BF), Ch06 (scattering channel, SSC), plus fluorescence channels Ch02, and Ch11 were activated for signal detection. Acquisition settings were the same in all conditions. Single cells were separated from debris and aggregates in the BF channel using the IDEAS features aspect ratio and area. Single color-stained controls were used for compensation, and non-stained and isotype ab control samples were used for autofluorescence or non-specific background. The data were analyzed with IDEAS^®^ software (v6.2.188.0).

### 2.5 Assessment of the activation of transcription factors of NFκβ and NFAT

The Jurkat reporter cells carry genetic reporter constructs, where the activation of NFκB, NFAT and AP-1 can be detected by measuring the expression of eCFP, eGFP and mCherry, respectively (Jutz et al., 2016). Due to the lack of a yellow laser in our FACSariaII and DxFLEX instruments, we were unable to analyze the activation of the AP-1 mCherry reporter gene. Hence, we refer to these triple reporter cells as Jurkat reporter cells and utilize the NFκB-eCFP and NFAT-eGFP reporter genes forour analysis in this study. To detect intracellular signaling response to target cell engagement, Jurkat reporter cells expressing the different FiCAR versions were cocultured with Nalm-6 B cells at a ratio of 1:1 for 24 h. The cells were collected and washed once at 400 g 5min with cold FACS buffer, fixed with 1% PFA for 30min and the samples were assessed with a flow cytometer (BD FACSAria IIU, software: FACSDiva™ v8.0.1, BD Biosciences) and data was analyzed with FlowJo^®^ (v10, BD Biosciences) software.

### 2.6 Live cell imaging by cell IQ high content screening fluorescent microscopy

48-well flat-bottom plates (VVR, cat#734-2780) were coated with poly-L-lysine (Merck cat# P4832-50 ML) for 2 h at room temperature. The plate was washed once with PBS and seeded with the mock LV and Jurkat reporter cells expressing FiCAR1 (7.5 × 10^5^) and kept in the cell incubator overnight. The next day the plate was washed once to remove the unbound cells and then 5 × 10^5^ Nalm-6 cells were added to the wells and monitored for the next 22 h. For live-cell imaging, the plate was covered with a Cell-Secure lid (Chip-Man Technologies, Tampere, Finland) containing ventilation filters and placed in the Cell-IQ incubator. Cell-IQ Imagen software (software version 4.1.0) was used for configuring and monitoring imaging, using the instrument’s basic settings. Pictures were taken at 5-min intervals. A green LED was used for phase contrast and a 465 nm LED to detect GFP expression. The captured phase contrast and fluorescence images were analyzed with Cell-IQ analyzer software (version 4.4.0). Videos were generated using ImageJ Fiji software.

#### 2.7 Cytotoxicity and degranulation assays

To measure the cytotoxic efficacy of the FiCAR variants, Jurkat reporter cells and CAR-expressing T cells from two individual healthy donors were cocultured with Nalm-6 luc^+^ target cells at several effectors: target (E:T) ratios for 16-18 h. Then luciferin reagent (ONE-Glo luciferase reagent, Promega, Madison, USA) was added and the live target cells were quantified with CLARIOstar Plus Microplate Reader, Software version 5.20 R5 (BMG Labtech). For the detection of target cell-induced degranulation of Jurkat reporter cells, the reporter cells expressing FiCARs, and control cells were cocultured with Nalm-6 target cells at a 1:1 ratio for 4 h in the presence of degranulation marker lysosomal-associated membrane protein 1 (CD107a) antibody (PE-conjugated, clone H4A3, BD Biosciences) and GolgiStop™ protein transport inhibitor (BD Biosciences). After incubation, the cells were fixed and the surface expression of CD107a in the effector cells was measured by flow cytometer.

### 2.8 Sample preparation for MS and analyses

FiCAR1-expressing Jurkat reporter cells were used as effector cells and mock LV-transduced Jurkat reporter cells were used as a negative control. Effector cells were stimulated using NIH/3T3 cells expressing human truncated CD19. The surface expression of CD19 on NIH/3T3 and FiCAR1 on Jurkat reporter cells was checked with flow cytometry before running the MS. First, CD19-expressing NIH/3T3 target cells were grown to confluence in 10 cm^2^-well dishes. The unbound cells were washed off with PBS and 10 million FiCAR1-or mock LV-expressing Jurkat reporter cells were added to the plate and incubated for 1 h. Next, the effector cells were collected with ice-cold buffer containing PBS (Invitrogen cat# A12856) and 25 mM EDTA (Fisher Scientific, versene cat# 15040066) and passed through a 30 µM filter (Miltenyi, cat#130-041-407). The cells were spun down at 500 g for 15 min and snap frozen and stored at −80°C for MS analyses. Two replicates for each sample were collected.

Cell lysis and protein denaturation were done by sonication in 8 mol/L urea (Amresco, Solon, OH, USA). Insoluble cell debris was removed by centrifugation at 21,000 *g* for 15 min at room temperature, and the samples were diluted to <1.5 mol/L urea with ammonium bicarbonate (AMBIC; Sigma-Aldrich). Before digestion, the protein samples were reduced with 5 mM Tris (2-carboxyethyl) phosphine (TCEP; Sigma-Aldrich) for 20 min at 37°C and then alkylated with 10 mM iodoacetamide (IAA; Sigma-Aldrich) for 20 min at room temperature in the dark. Sequencing Grade Modified Trypsin (Promega) was then used at a 1:100 enzyme-substrate ratio and the samples were incubated overnight at 37°C. After digestion, the samples were desalted with C18 macro-spin columns (Nest Group). The macro-spin columns were first conditioned by centrifuging 200 μL of 100% acetonitrile (CAN) through at 55 g, followed by 200 μL of water. The column was then equilibrated twice with 200 μL of buffer A (0.1% trifluoroAcetic acid, TFA, 1% ACN). Samples were then added 100 μL at a time and washed twice with 200 μL of buffer A. Finally, the sample was released with 3 × 200 μL of elution buffer (80% ACN, 0.1% TFA).

Phospho-peptide enrichment was performed using immobilized metal ion affinity chromatography with titanium IV) ion (Ti4+-IMAC). The IMAC material was prepared by following the steps of the protocol published previously (Zhou et al., 2013b). For enrichment of phosphopeptides, the Ti4+-IMAC beads were loaded onto GELoader tips (Thermo Fisher Scientific). The material was then conditioned with 50 μL of conditioning buffer (50% CH3CN, 6% TFA) by centrifuging at 150 g until all of the buffers had passed through. The protein digests were dissolved in a loading buffer (80% CH3CN, 6% trifluoroacetic acid (TFA)) and added into the spin tips and centrifuged at 150 g until all had passed through. The columns were then washed with 50 μL of wash buffer 1 (50% CH3CN, 0.5% TFA, 200 mM NaCl), followed by 50 μL of wash buffer 2 (50% CH3CN, 0.1% TFA), and finally, the bound phospho peptides were eluted with 10% ammonia, followed by a second elution with elution buffer (80% CH3CN, 2% FA). Samples were then dried in a vacuum centrifuge and reconstituted in a final volume of 15 μL in 0.1% TFA and 1% CH3CN. The dried peptides were reconstituted in 30 µL Buffer A (0.1% (vol/vol) TFA and 1% (vol/vol) acetonitrile (ACN) in HPLC water). Samples were further diluted 1 + 19 µL with HPLC water containing 0.1 vol/vol% formic acid. The manufacturer’s instructions were followed to load into Evotips (Evosep).

#### 2.8.1 Liquid chromatography-mass spectrometry (LC-MS) analysis

The desalted samples were analyzed using the Evosep One liquid chromatography system coupled to a hybrid trapped ion mobility quadrupole TOF mass spectrometer (Bruker timsTOF Pro) *via* a CaptiveSpray nano-electrospray ion source. An 8 cm × 150 µm column with 1.5 µm C18 beads (EV1109, Evosep) was used for peptide separation with the 60 samples per day methods (21 min gradient time). Mobile phases A and B were 0.1% formic acid in water and 0.1% formic acid in acetonitrile, respectively. The MS analysis was performed in the positive-ion mode using data-dependent acquisition (DDA) in PASEF mode with DDA-PASEF-short_gradient_0.5s-cycletime -method.

Raw data (. d) were processed with FragPipe v16.0 utilizing MSFragger (Yu et al., 2020) against reviewed human entries of the UniProt KB database. Carbamidomethylation of cysteine residues was used as static modification. Amino terminal acetylation, oxidation of methionine, and phosphorylation of serine, threonine, or tyrosine were used as variable modifications. Trypsin was selected as enzyme, and maximum of two missed cleavages were allowed. Both instrument and label-free quantification parameters were left to default settings. Results from these steps are Spectral Counts (SC) values from peptides with FDR <0.01 from Philosopher.

For further analysis, the average intensity of two replicates were calculated for each peptide and peptides were mapped to corresponding proteins. Proteins having one or more phosphorylations were directed to KEGG pathway analysis using the DAVID bioinformatic tool (Chen et al., 2020). The KEGG database is generated by molecular level information which can be implied to predict in which pathways a particular gene or protein is enriched. Phosphorylation sites of certain interesting proteins were checked manually. To concentrate on the most significant changes in protein phosphorylation, only the appearance or disappearance of phosphorylation in FiCAR1 *versus* mock transduced samples was counted.

## 3 Results

Previously we designed a novel second generation CD19-targeting FiCAR1 where the spacer part of FiCAR backbone is derived from SIRPα, the scFv from FMC63 and the signaling domains are from CD28 and CD3ζ. We showed that the SIRPα backbone-based CARs evade deleterious interactions with Fc receptor-expressing cells *in vitro* and *in vivo* (Koski et al., 2022).

### 3.1 Modification of the structure of the FiCAR backbone

**FIGURE 1:**
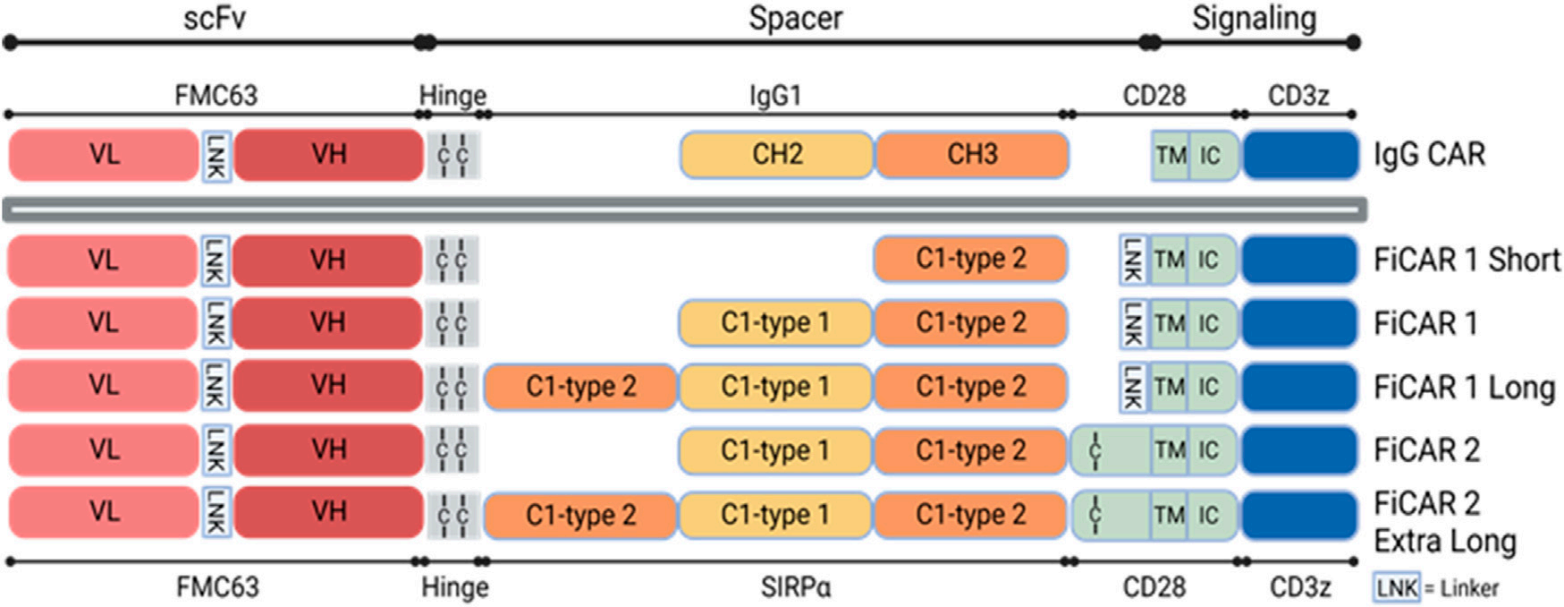
Schematic structures of different versions of CD19 targeting FiCARs. The antigen binding domain, scFv is from the FMC63 monoclonal antibody recognizing CD19. The spacer is derived from SIRPα C1-type1 and C1-type2 domains, the TM domain is from CD28, and the intracellular signaling domains are from CD28 and CD3ζ. In the IgG CAR, the spacer is derived from IgG1 CH2 and CH3 domains.

The physical dimensions of generic CAR molecules may limit their capability to interact with certain targets (Hudecek et al., 2015). Consequently, re-dimensioning of CARs to optimally engage such antigens may be required (Kuünkele et al., 2015; Watanabe et al., 2016). To tackle such problems and using FiCAR1 as a starting point, we designed CD19-targeted FiCARs containing SIRPα spacer of five different lengths and modifications. We believe that FiCARs of different physical dimensions may be useful in future experiments where accessibility to the target antigens in different tumors might be problematic. The FiCAR1 spacer comprises the Ig-like C1-type1 and C1-type2 domains of SIRPα. Subsequently, in the current study, we modified the length of the spacer by including only the Ig-like C1-type 2 domain (FiCAR1 S (short)), or by including an additional C1-type 2 domain (FiCAR1 L (long)) to the FiCAR1 structure. Most CARs are believed to exist on the T cell surface as disulfide-linked dimers (Van Der Stegen et al., 2015). However, the role of dimer formation in CAR function is relatively unexplored. Hence, in our earlier work, we designed FiCAR2, which in comparison to FiCAR1 has an additional short extracellular juxtamembrane sequence derived from CD28. The CD28 sequence contains the cysteine that dimerizes the native CD28 molecule (Evans et al., 2005), and we hypothesize that it may also form a cysteine bridge in FiCAR2 thereby potentially stabilizing the long molecule. The FiCAR2 XL (extra-long) incorporates three SIRPα-derived Ig-like domains and the CD28-derived juxtamembrane putative dimerizing domain (Figure 1).

### 3.2 The Jurkat reporter T cell line can be used as a tool to accelerate CAR development

**FIGURE 2:**
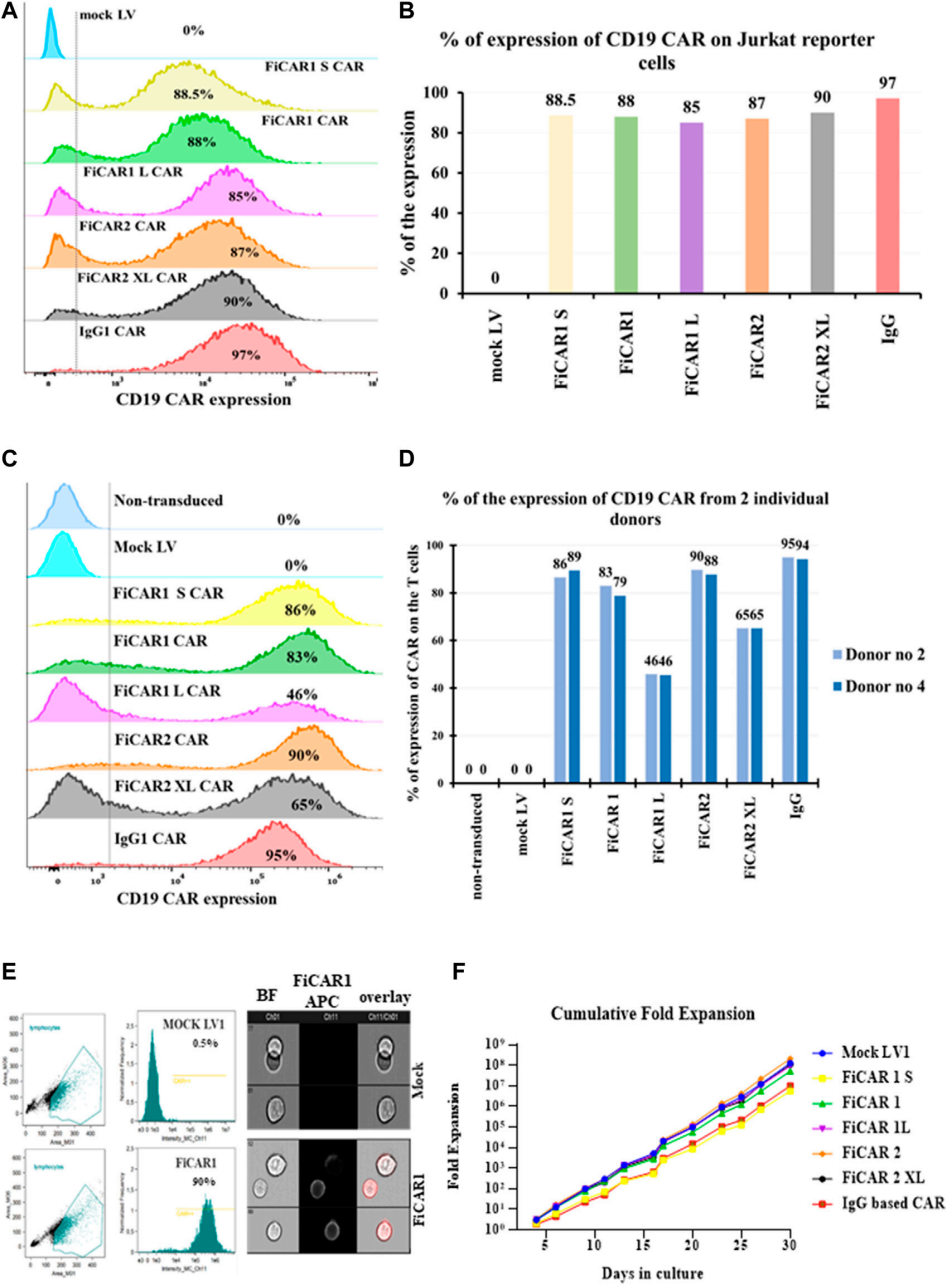
Cell surface expression and growth of Jurkat reporter cells expressing the different CD19 targeting FiCAR versions. **(A)** Cell surface expression of the FiCARs was measured by a flow cytometer using mock LV as a negative control and, a generic IgG CAR as a comparator. For the detection of the CARs, a CD19 CAR detection reagent was used. **(B)** A bar graph shows the expression of different modified CARs in Jurkat reporter cells. **(C)** The different versions of modified CD19-targeted FiCARs were transduced into primary T cells and the expression of CARs was assessed with flow cytometry. **(D)** A bar graph shows the expression of different modified CARs in T cells from two healthy donors. **(E)**The FiCAR1 expression was analyzed using imaging flow cytometry (Amnis). The gating strategy of cell populations and histograms showing the expression of CD19 binding in FiCAR1 and mock transduced Jurkat reporter cells are shown in the left panels, fluorescent images of FiCAR1 expressing cells and mock transduced cells are seen in the right panels. Ch (channel) 01 is for the bright field (BF) and Ch11 is for APC fluorochrome. **(F)** Growth curves of Jurkat reporter cells transduced with FiCARs, IgG CAR and mock viruses. The viability of the cells was assessed with trypan blue and TC-20 (Bio-Rad) automated cell counter. Cultures were monitored every 2-3 days.

When designing and testing novel FiCARs there is a need for reductionist models to produce, express and evaluate the modified CARs to expedite discovery. For this purpose, we have used a Jurkat reporter cell line for evaluating the functions of different FiCAR variants *in vitro*. The Jurkat reporter cells were transduced with LVs encoding the different variants of the CAR genes. A LV vector carrying no transgene (mock) was used as a negative control and an IgG-based CAR (second generation) (Kaartinen et al., 2017) as a comparator. FiCARs were expressed on 85%-97% of the Jurkat reporter cells (Figures 2A,B), as assessed by flow cytometry on day six post-transduction and as also seen in primary T cells from two donors (Figures 2C,D). We were also curious to know whether FiCAR1 was expressed evenly on the cell surface or if there was aggregation or internalization of the CARs without activation of the cells. To answer this question, we analyzed the stained reporter cells expressing FiCAR1 with the Amnis imaging flow cytometer. Immunofluorescence pictures from the cytometer revealed that FiCAR expression was even on the surface of reporter cells and mock cells showed no expression (Figure 2E). The histogram showed that FiCAR1 was expressed in 90% of transduced cells and reactivity was detected in 0.5% of mock cells (Figure 2E).

We also followed the growth of the Jurkat reporter cells carrying different FiCARs for 30 days and saw exponential cell expansion in culture (Figure 2F). No statistically significant differential effects on growth velocity were observed among the different modified FiCARs, except for a non-significant trend toward slower growth in FiCAR1 S and IgG CAR-expressing cells. We noticed a reduced expression of the different variants of FiCARs in Jurkat reporter cells after the fourth week of subculturing the cells (data not shown).

### 3.3 Intracellular activation of NFκβ and NFAT induced by the various FiCAR modifications targeting CD19-expressing cancer cells

**FIGURE 3:**
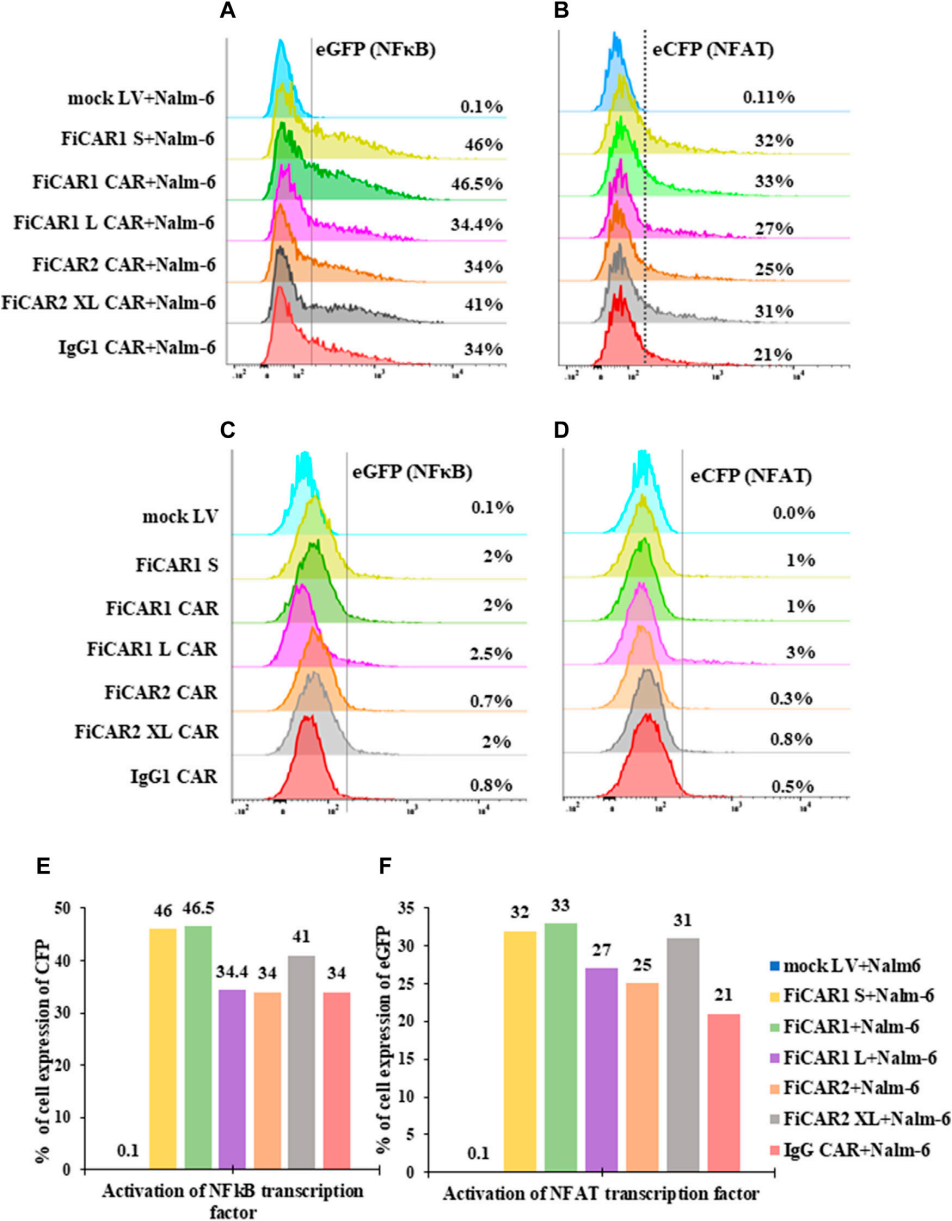
Activation of NFkB and NFAT transcription factors upon target cell engagement. (**A–D)** Jurkat cells, expressing the CD19 targeted FiCARs, IgG CAR and mock LV were cocultured with CD19 positive Nalm-6 cells at a ratio 1:1 for 24 h. Control cells were cultured without Nalm-6 cells. The expression of eCFP and eGFP as indicator of NFkB and NFAT activation, respectively, were analyzed by the flow cytometry. **(E, F)** Bar graphs represent the % of reporter cells expressing eCFP and eGFP.

An ideal CAR will bind the target antigen and convey effective intracellular signals *via* the CD28 and CD3ζ domains to induce T cell activation. We used the E6.1 Jurkat reporter cells to interrogate the activation of NFκβ and NFAT simultaneously by measuring the expression of eCFP and eGFP by flow cytometry. Following coculture of the Jurkat reporter cells expressing the modified FiCARs with B cells at a ratio of 1:1 for 18 h, we detected activation of both NFκβ in 34%-46% (Figures 3A,E) and NFAT in 21%-32% (Figures 3B,F) in FiCAR expressing cells, but not in Jurkat cells transduced with an empty virus. No antigen-independent activation of NFκβ or NFAT was induced by any of the CD19-targeting FiCARs (Figures 3C,D). These data indicate that the fluorescence signal can be used as an indicator of the presence of functional FiCARs on the Jurkat reporter cells and that the different CAR constructs effectively institute intracellular signaling directing T cell function upon target cell engagement. To better understand the timescale of the effects of FiCAR activation while targeting B cells, we performed live cell imaging with a Cell-IQ high throughput microscope. During coculture of FiCAR1 and Nalm-6 cells the intensity of the eGFP fluorescent protein, which is driven by NFAT activation started to appear after 3 h, whereas no expression of eGFP was seen from coculture of mock cells with Nalm-6 cells (Supplementary Figure S1A (video), C, D).

### 3.4 Jurkat reporter line can be used to measure the killing efficacy of the novel FiCAR variant

**FIGURE 4:**
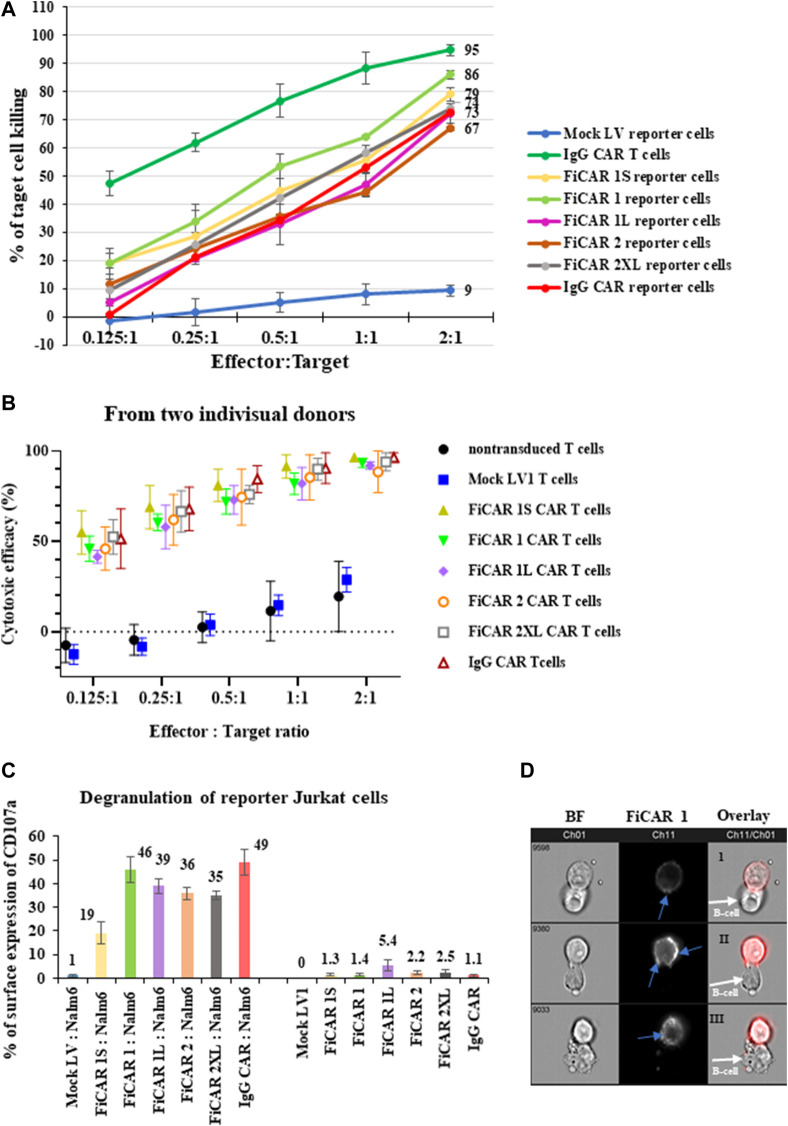
Cytotoxicity against CD19^+^ Nalm-6 cells of Jurkat reporter cells expressing different versions of CD19 targeting FiCARs. **(A)** Primary T Cells expressing IgG CAR and mock LV transduced reporter cells were used as positive and negative controls, respectively. The effector cells were cocultured with luc + target cells at various E: T ratios for 18 h and the remaining live target cells were quantified by measuring luminescence. **(B)** Efficacy against Nalm-6 cells of primary T cells transduced with different modified CD19 targeting FiCARs. Mock LV transduced and non-transduced T cells were used as a negative control. The graph shows the mean, and high and low values (n = 2). **(C)** Degranulation of FiCAR expressing reporter cells in response to Nalm-6 cells was measured by analyzing the surface expression of CD107a after 4 h of coculture of effectors with target cells at a ratio of 1:1. Effector cells cultured without target cells were used as control. The SD was calculated from three individual experiments. **(D)** Jurkat reporter cells were cocultured with target cells for 4 h, then labeled with CD19 CAR detection reagent and analyzed with an Amnis flow cytometer. Ch01 shows bright field images from the reporter cells expressing FiCAR1 and B cells, Ch11 shows only the immunofluorescence images, and in Ch11/Ch01 an overlay of BF and immunofluorescence images are shown. Blue arrows in the FiCAR1 panel point to concentrations of CAR on the effector cell surface. White arrows in the overlay panel indicate target cells.

The previous data show that FiCARs were successfully expressed on the Jurkat reporter cells and CAR-expressing Jurkat cells became activated when they encountered CD19^+^ B cells. Next, we asked the question whether Jurkat cells expressing these FiCAR were also able to kill B cells. The effector cells were cocultured with luciferase-positive Nalm-6 cells at various E: T ratios for 18 h, followed by quantification of the remaining live cells by measuring luciferase activity. All Jurkat reporter cells expressing the FiCARs, and the control IgG CAR displayed cytotoxicity against the target B cells (Figure 4A). Jurkat cells equipped with FiCAR1 were somewhat more effective killers than cells transduced with other FiCAR versions. Furthermore, primary T cells armed with different versions of CD19 targeted CARs displayed potent cytotoxicity against Nalm-6 cells and no significant differences were seen in efficacy between cells prepared from two donors (Figure 4B). As expected, the efficacy of killing by Jurkat reporter cells is less than that of primary T cells expressing CARs.As the FiCAR-expressing Jurkat reporter cells were able to kill the B cells, we were interested to investigate if we could detect evidence of degranulation by the effector cells following an encounter with target cells. Degranulation of lytic granules occurs in T cells upon activation by antigen (Betts and Koup, 2004). Indeed, after a 4-h coculture of the FiCAR expressing Jurkat cells with Nalm-6 cells, robust surface expression of CD107a was detected on the reporter cells carrying modified FiCARs, while mock transduced Jurkat cells in coculture and FiCAR expressing Jurkat cells without coculture with targets did not express CD107a (Figure 4C). We observed surface CD107a expression in 46% of activated reporter cells expressing FiCAR1 which likewise displayed the highest killing efficacy. The expression of surface CD107a in the Jurkat reporter cells expressing the other modified FiCAR variants protein varied between 19% and 35%. We were also curious to know whether there is a re-localization of the FiCAR molecules on the Jurkat cell surface in response to target cell engagement. To document this, we analyzed FiCAR1 expressing Jurkat reporter cells after 4 h of coculture with Nalm-6 target cells using the Amnis imaging flow cytometer. Interestingly, while Jurkat cells that had not been in contact with target cells displayed an even distribution of FiCAR1 molecules over the cell surfaces (Figure 2E), after coculture with CD19^+^ target cells the FiCAR1 molecules were concentrated in distinct clusters. Furthermore, FiCAR1 molecules were also clustered at sites of effector-target cell contact (Figure 4D). In summary, these data show that reporter cells with different modified FiCARs were able to kill target cells, and importantly the reporter cells can be used to probe CAR function.

### 3.5 The functionality of FiCARs aimed at other targets can be interrogated using the Jurkat reporter cell line

**FIGURE 5:**
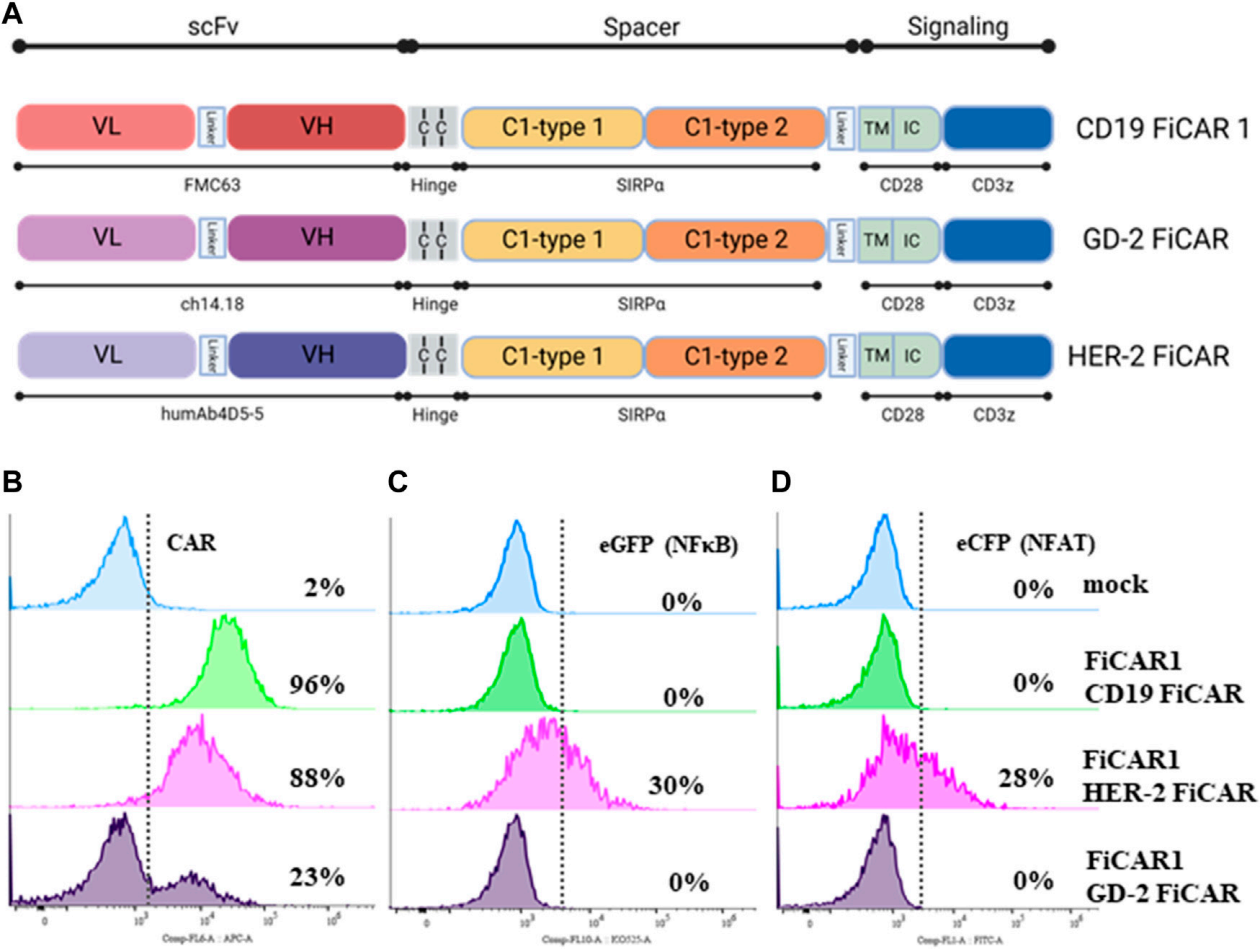
Structure of CD19, GD2 and HER-2 FiCARs and their expression on Jurkat reporter cells. **(A)** The schematic structures of the CD19, GD2 and HER-2 FiCARs. **(B–D)** Jurkat reporter cells were transduced with mock LV, or LV’s carrying CD19, HER-2, GD-2 FiCARs, stained with conjugated SIRPα ab and analyzed with a DxFLEX cytometer for **(B)** FiCAR expression, **(C)** eCFP expression (as a marker of NFκB activity), and **(D)** eGFP expression (as a marker of NFAT activity).

As we found that the Jurkat reporter line is a good initial tool for evaluating different variants of the CD19 targeting FiCAR, we extended our studies to find active CARs against solid tumor targets such as breast cancer and neuroblastoma. For this purpose, we engineered FiCAR1 constructs where the scFv sequence of the FMC63 monoclonal antibody was replaced with corresponding sequences of the humAb4D5-5 and Ch14.18 antibodies recognizing HER-2 and GD2, respectively (Figure 5A), and transduced them into Jurkat reporter cells. SIRPα-Cy5 ab was used in these experiments for the detection of FiCARs whether targeting CD19, HER-2 or GD2. A high-level expression of CD19 FiCAR1 (96%), HER-2 FiCAR1 (88%) and moderate expression of GD2 FiCAR1 (23%) were observed using flow cytometry (Figure 5B). Interestingly, antigen-independent activation was observed in Jurkat reporter cells expressing the HER-2 targeted FiCAR as there was activation of NFκβ (30%) and NFAT (28%) in resting reporter cells, a phenomenon that was absent in the CD19 and GD2 targeting FiCARs (Figures 5C,D). Reporter Jurkat cells expressing the HER-2 targeted FiCAR displayed ample cytotoxicity against SKBR3 breast cancer cells (Supplementary Figure S2D).

The THP monocyte cell line served as a positive control for the SIRPα ab (Supplementary Figure S2B) as monocytes commonly express the SIRPα protein (Ha et al., 2013). We observed that this monoclonal SIRPα antibody reacted with such FiCARs that contained both the SIRPα Ig like C1-type1 and the C1-type2 domains but did not recognize FiCAR1 short that incorporates only a single C1-type2 domain. While this is not a formal proof, it suggests that the SE12B6A4 antibody recognizes the C2-type1 domain in SIRP-α (Supplementary Figure S2A, C).

### 3.6 Intracellular signaling induced by target engagement of Jurkat reporter cells carrying FiCAR1

It has been well studied that activation of TCR leads to the phosphorylation of ITAMs (immunoreceptor tyrosine-based activation motifs) on CD3ζ then conveying the intracellular activation signal *via* the phosphorylation of various proteins like LCK, ZAP70 and SLP-76. This finally leads to the activation of the nuclear transcription factors NFκβ, NFAT and AP-1 which play an important role in T cells’ proliferation, activation, and persistence (Bhattacharyya and Feng, 2020). It has been reported that also CARs signal through endogenous TCR engaging proteins (Salter et al., 2018; Wu W. et al., 2020). To understand better the functions of FiCAR1 in Jurkat reporter cells we performed phosphoproteomic analyses by MS in FiCAR1 expressing Jurkat cells that were activated by CD19^+^ target cells. Mock LV transduced cells that were treated similarly were used as a negative control. In this experiment, we used CD19 expressing NIH/3T3 mouse fibroblast cells as the activating target cells because signals from possible contaminating murine proteins could be excluded in the analysis of the MS data. The expression of CD19 on NIH/3T3 and the expression on FiCAR1 from Jurkat reporter cells was ascertained by flow cytometry before the experiment (Supplementary Figure S3A, B). The effector cells were cocultured with the target cells for 1 h, after which the samples were collected and frozen for later MS analysis. In coculture the Jurkat reporter cells expressing FiCAR1 aggregate around the target cells whereas such aggregation was not seen in mock transduced Jurkat cells (Supplementary Figure S3D, E). This aggregation is suggestive of active engagement of the CD19^+^ target cells by FiCAR1-carrying Jurkat cells.

**FIGURE 6:**
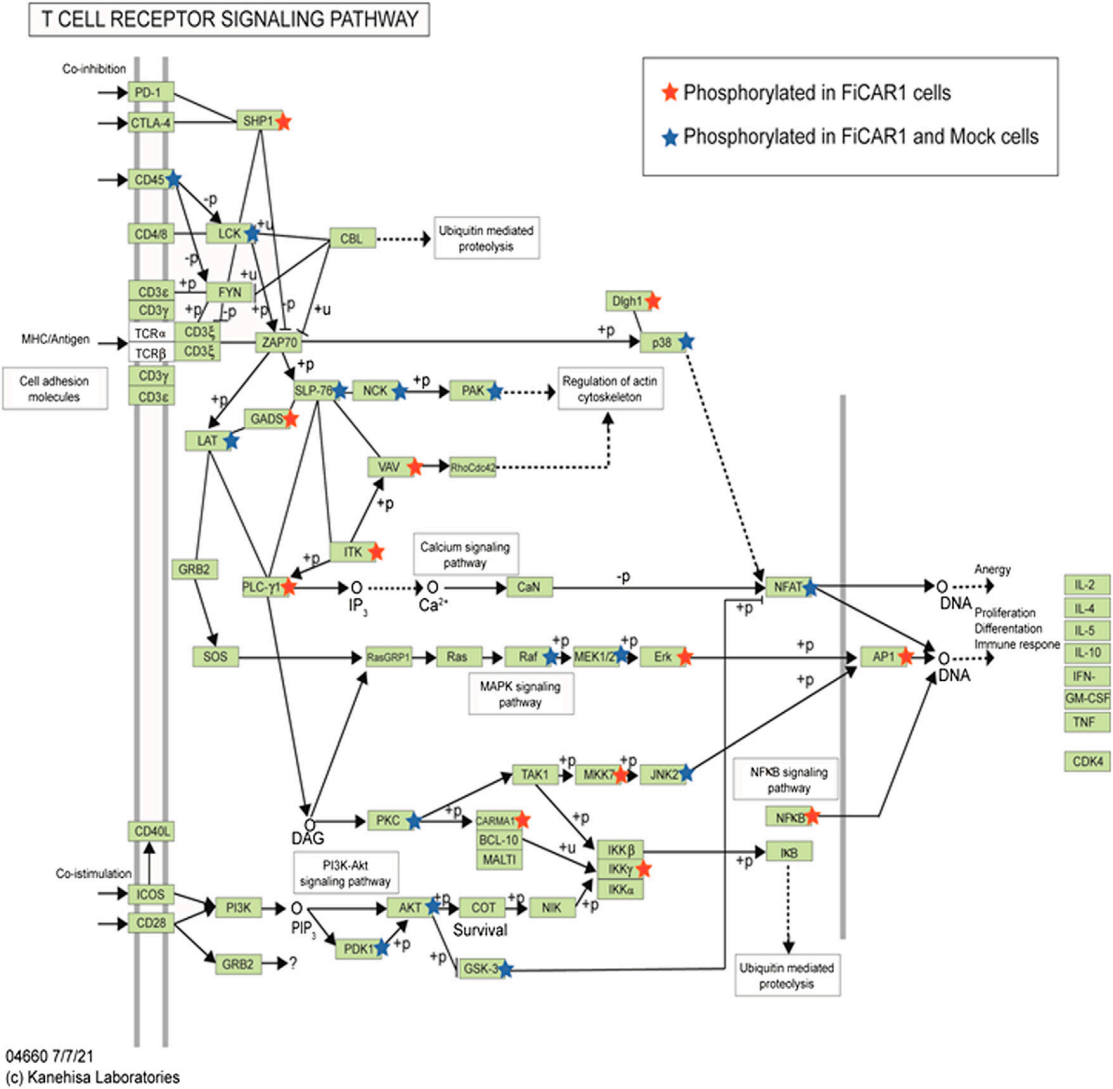
The T Cell receptor signaling pathway (information generated by KEGG, DAVID tool). Phosphorylation of the proteins in this pathway is indicated with stars. Red stars indicate phosphorylated proteins that were found in activated FiCAR1 samples; blue stars indicate phosphorylated proteins that were found in both mock and FiCAR1 cells.

**FIGURE 7:**
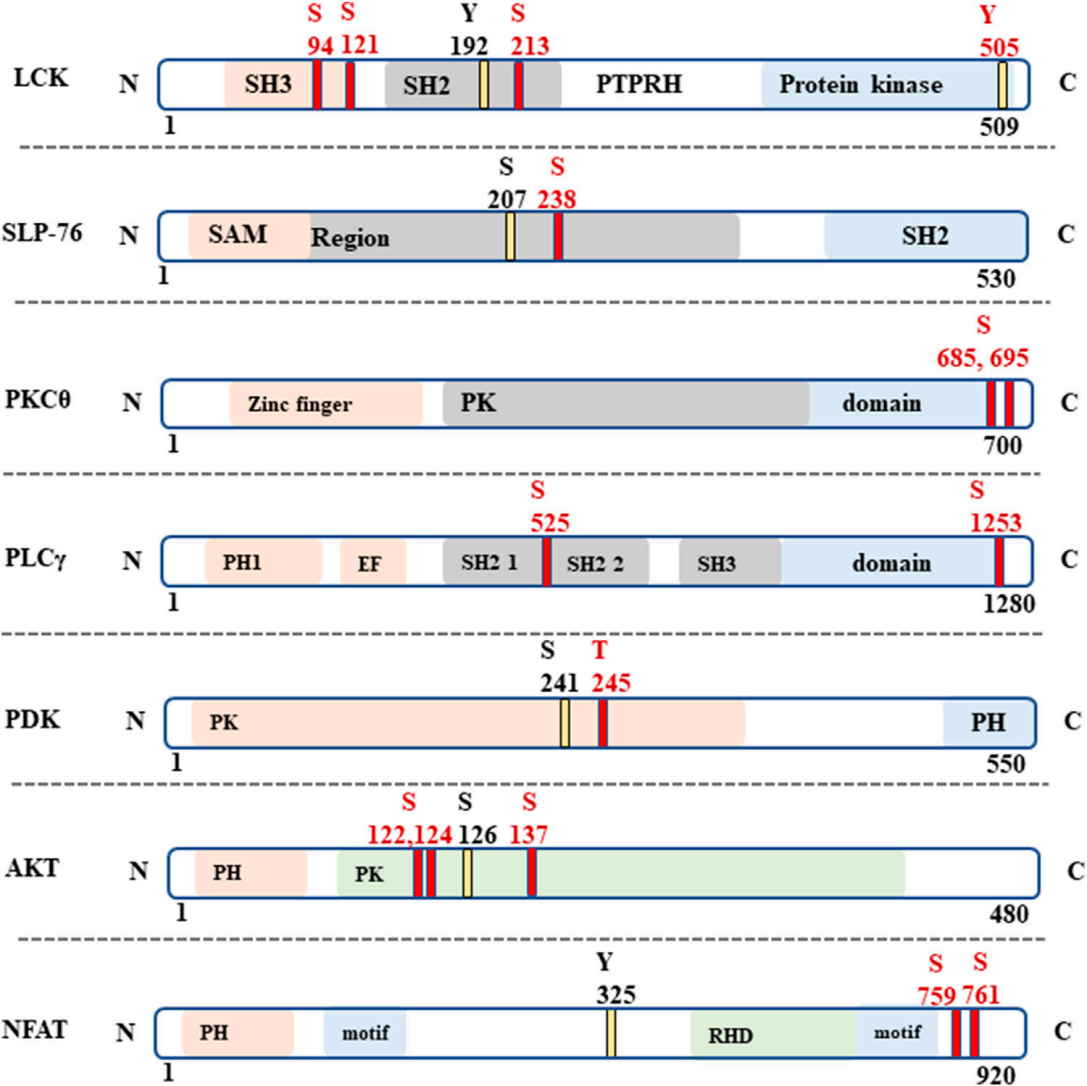
Specific phosphorylated amino acid (aa) in signaling proteins. The phospho amino acids (aa) which were found in both activated FiCAR1, and mock samples are indicated with yellow lines and the phospho-aa which were found only in activated FiCAR1 Jurkat cells are indicated with red lines. S=Serine, Y = Tyrosine and T = Threonine.

In total 7,985 peptides were identified, of which 6,585 had one or more phosphorylations (mass shift 79.9663) either in serine S), tyrosine Y) or threonine T) (Supplementary Table S1). Of total peptides, 2655 were found in mock samples and 7,363 in FiCAR1 samples and from these 2297 and 6,133 were phosphorylated, respectively. Peptides were derived from 2271 different proteins, of which 1,082 were detected in the Mock and 2220 in FiCAR1 samples (Supplementary Table S1). From these 972 proteins had one or more phosphorylations in Mock samples and 1914 in FiCAR1 samples. KEGG enrichment analysis by the DAVID bioinformatic tool (Chen et al., 2020) of phosphorylated proteins revealed significant enrichment in several intracellular pathways (Table 1). Comparison of KEGG pathway enrichment between mock and FiCAR1 transduced samples showed that certain pathways, such as the T cell receptor signaling pathway and the MAPK signaling pathway, were significantly more enriched in the analyses of the phosphorylated proteins in FiCAR1 transduced than in mock transduced cells (Table 1). This indicates an increased phosphorylation level of these pathways in FiCAR1 samples. A closer investigation of pathway maps clearly shows the difference in the number of phosphorylated pathway members (Figure 6; Supplementary Figure S4). In addition, we have investigated manually the single phosphorylation sites of a few important key players in FiCAR1-induced T cell signaling. These include tyrosine protein kinase (LCK), SH2 domain-containing leukocyte protein of 76 (SLP-76), 1-phosphatidylinositol 4,5-bisphosphate phosphodiesterase gamma-1 (PLCγ1), NFAT and protein kinase C theta (PKCθ), 3-phosphoinositide-dependent protein kinase 1 (PDK), serine-threonine protein kinase (AKT). In these proteins, we observed that certain amino acids were phosphorylated only in activated FiCAR1 cells and not in mock cells (Figure 7). These data show that there are significant changes in the phosphorylation levels of intracellular signaling molecules between FiCAR1 expressing and mock Jurkat cells upon target cell engagement.

**TABLE1:** KEGG pathway analyses using DAVID tools.

Category	Term	Description	Count Mock/FiCAR1	*p*-value/Mock	*p*-value/FiCAR1	Comments
KEGG_PATHWAY	hsa04660	T cell receptor signaling pathway	22/34	3.7E-08	7.4E-10	↑FiCAR
KEGG_PATHWAY	hsa04530	Tight junction	18/36	0.005	2.1E-05	↑FiCAR
KEGG_PATHWAY	hsa04370	VEGF signaling pathway	10/16	0.002	5.30E-04	↑FiCAR
KEGG_PATHWAY	hsa05166	Human T Cell leukemia virus 1 infection	21/50	0.005	6.0E-05	↑FiCAR
KEGG_PATHWAY	hsa04010	MAPK signaling pathway	24/52	0.02	5.6E-05	↑FiCAR
KEGG_PATHWAY	hsa04510	Focal adhesion	16/37	0.08	3.1E-02	↑FiCAR
KEGG_PATHWAY	hsa05235	PD-L1 expression and PD-1 checkpoint pathway in cancer	10/22	0.03	1.4E-04	↑FiCAR
KEGG_PATHWAY	hsa04810	Regulation of actin cytoskeleton	18/37	0.04	0.001	↑FiCAR
KEGG_PATHWAY	hsa04666	Fc gamma R-mediated phagocytosis	10/22	0.06	4.9E-04	↑FiCAR
KEGG_PATHWAY	hsa04066	HIF-1 signaling pathway	0/21	no	0.005	↑FiCAR
KEGG_PATHWAY	hsa04670	Leukocyte trans-endothelial migration	0/20	no	0.001	↑FiCAR
KEGG_PATHWAY	hsa04657	IL-17 signaling pathway	0/22	no	5.9E-04	↑FiCAR
KEGG_PATHWAY	hsa04668	TNF signaling pathway	0/24	no	5.9E-04	↑FiCAR
KEGG_PATHWAY	hsa04062	Chemokine signaling pathway	0/30	no	0.01	↑FiCAR
KEGG_PATHWAY	hsa04015	Rap1 signaling pathway	0/33	no	0.01	↑FiCAR
KEGG_PATHWAY	hsa04620	Toll-like receptor signaling pathway	0/18	no	0.02	↑FiCAR
KEGG_PATHWAY	hsa04014	Ras signaling pathway	0/33	no	0.04	↑FiCAR
KEGG_PATHWAY	hsa04064	NF kappa B signaling pathway	0/17	no	0.05	↑FiCAR

KEGG: Kyoto Encyclopedia of Genes and Genomes. MS, data in mock and reporter cells expressing FiCAR1 were uploaded into DAVID, tools Enriched ↑ or not ↓pathways in activated FiCAR1 cells were shown by comparing the *p*-value between mock and FiCAR1 cells.

## 4 Discussion

Adoptive cell therapy with CAR-modified T cells has made great strides in the treatment of hematological cancers, as already five hematological CAR T cell therapies have been approved by the US Food and Drug Administration (Pan et al., 2022). Nevertheless, many challenges still need to be tackled when it comes to liquid and especially solid cancers, including optimizing CAR T cell manufacturing, scarcity of tumor-specific antigens, inefficient CAR T cell trafficking to the target site and also antigen leakage (D’Aloia et al., 2018; Pan et al., 2022). Previously, we used an IgG1-based CAR where the spacer was derived from IgG1 that was functional *in vitro* (Kaartinen et al., 2017; Dufva et al., 2020). However, the IgG1-CH2 domain interacts with Fc receptor-expressing cells *in vivo* leading to CAR T cell dysfunction (Hombach et al., 2010; Almåsbak et al., 2015). To overcome this problem, we developed a novel FiCAR backbone where the spacer is derived from SIRPα, which avoids off-target interactions *in vitro* and *in vivo* (Koski et al., 2022). Optimizing CAR-antigen interactions may provide avenues for improving CAR T cell efficacy. The ability to modulate the length of the CAR spacer is necessary for developing functional CARs against certain solid tumor target antigens (Hudecek et al., 2015; Kuünkele et al., 2015). Here we have made five different versions of the FiCAR backbone where the length of the spacer is varied FiCAR1, FiCAR1 S, FiCAR1 L, FiCAR2 and FiCAR2 XL (Figure 1). We posit that the ability to adjust the physical dimensions of CARs will be useful when targeting various antigens in different solid tumors.

To interrogate the functionality of the different FiCAR versions, we used the well-established CD19 system, with CD19 expressing Nalm-6 Bcells as targets. However, analyzing multiple novel CARs is slow and laborious. To expedite the work, we made use of a triple parameter Jurkat T cell line which was developed for assessing the functions of various receptors related to T cell function (Jutz et al., 2016; Rosskopf et al., 2018; Müller et al., 2020; Battin et al., 2022). Using the reporter cells, we show that all FiCAR versions with different spacer lengths are expressed on the surface of 85%-97% of the cells (Figures 2A, B, E). We also show that the different modified FiCAR variants in Jurkat reporter do not impair cell survival during a 30 days expansion (Figure 2F). Only minor non-significant differences in the effect of the different FiCARs on cell growth were observed. All of these CD19-targeted FiCARs were also expressed in primary T cells (Figure 2C, D). CD19-targeted FiCARs induced robust activation of NFκβ and NFAT when Jurkat reporter cells engaged Nalm-6 cells whereas no activation of NFκβ and NFAT was seen in mock LV transduced reporter cells or FiCAR-expressing reporter cells cultured without targets (Figures 3A–F). Cell-IQ high throughput live cell imaging of FiCAR1 reporter cells cocultured with Nalm-6 cells revealed NFAT activation beginning after 3 h, as revealed by the appearance of eGFP fluorescence. (Supplementary Figures S1C, D). The appearance of the fluorescing protein can be seen on video (Supplementary Figure S1A). The data also indicate that the different CD19 targeting FiCAR constructs are not self-activated i.e. there is no tonic signaling by these CARs.

Because we detected good expression and cell activation by the different FiCAR versions, we were interested to see whether they would also induce directed cytotoxicity by the Jurkat reporter cells. FiCAR-expressing Jurkat reporter cells kill CD19^+^ B cells significantly more efficiently than mock LV-transduced Jurkat reporter cells (Figure 4A). Assessment of killing efficacy in primary T cells that were modified with the same CD19 FiCAR constructs corroborated the findings obtained with Jurkat reporter cells (Figure 4 A, B). We were also able to document the killing of Nalm-6 cells by FiCAR1 expressing Jurkat cells using the Cell-IQ^®^ automated cell culture and analysis system (Supplementary Figure S1B). The efficacy of killing by this CD4-positive T cell line is less than that displayed by primary T cells expressing CARs (Figures 4A,B). CD8^+^ T cells have traditionally been considered responsible for cytotoxicity, as even without CD4^+^ T cells MHC class I-restricted T cell receptor (TCR) transgenic mice maintained their anti-tumor activity (Hanson et al., 2000). Yet, the importance of CD4^+^ T cells in cancer elimination *via* secretion of effector cytokines such as interferon-γ (IFNγ) and tumor necrosis factor-α (TNFα) has also been reported (Tay et al., 2021). Furthermore, CD4^+^ T cells can eradicate the tumor cells even in the absence of CD8^+^ T cells (Haabeth et al., 2014). Accordingly, we detected high expression of surface CD107a by target-activated Jurkat reporter cells expressing FiCARs (Figure 4B). As an increased expression of CD107a on activated NK-cells and T cells parallels an increase in their cytotoxicity, it has been suggested that CD107a expression is a sensitive marker for their cytotoxic potential (Aktas et al., 2009). We also observed the aggregation of FiCAR1 receptors in activated Jurkat reporter cells (Figure 4C) by imaging flow cytometry which is an important initial step for signaling by CARs (Wu L. et al., 2020).

Following these results, we tested whether the FiCAR backbone can be engineered to target other antigens such as HER-2 and GD2. Constructs recognizing HER-2 and GD2 were successfully transduced into Jurkat reporter cells (Figure 5B). Interestingly, Jurkat reporter cells expressing HER-2 displayed activation of NFκβ and NFAT (Figures 5C,D) even without engagement with target cells. Preliminary results indicate that this particular anti-HER-2 FiCAR displays tonic signaling also when transduced into primary T cells, with detrimental effects on their function (Manar Elmadani, unpublished results).

Synthetic receptors have been developed to mimic the T cell signaling cascades to treat cancers efficiently. While CAR T cell treatment often cures B cell malignancies, sometimes it causes life-threatening complications like cytokine release syndrome (CRS) and neurotoxicity (Gust et al., 2017; Hay et al., 2017). Both efficacy and toxicity are affected by the activation of intracellular signaling pathways mediated by CAR engagement (Wu W. et al., 2020). Hence, we were interested to know how the FiCAR1 molecule instigates intracellular signaling upon interaction with the target cell. We show that the activation of FiCAR1 leads to the phosphorylation of LCK, which can phosphorylate CD3ζ. In the phospho-peptide MS analysis data, the phosphorylation of LCK was found both in mock and FiCAR1 transduced cells, but interestingly three phosphoserines (S94, S121 and S213) were only found in activated FiCAR1 but not in mock transduced cells. Tyrosine Y) 192 phosphorylation, however, was found in both conditions (Figure 7). The serine and threonine kinase activity are necessary for T cell activation (Mayya et al., 2009). It has been shown previously that increased basal phosphorylation of the CAR CD3ζ chain and CAR-associated LCK contribute to the rapid kinetics and stronger signal strength of CD28/CD3ζ CARs (Salter et al., 2018). S207 phosphorylation in SLP-76 was found in mock transduced and (activated) FiCAR1 cells and interestingly phospho S 207 in SLP-76 only can be found in resting human platelets (Zahedi et al., 2008). In addition, there is another phospho S238 that was found only in FiCAR1 cells (Figure 7), which might be more crucial for the signaling. Phosphorylation of PLCγ1 was only seen in FiCAR cells and it plays an important role in the activation of the Ca^2+^ signaling pathway in T cells. PLCγ1 is also an important factor for the activation of PKCθ. S695 phosphorylation in the activation loop of PKCθ is reported to be crucial for NFκB induction (Liu et al., 2002) and phosphorylation of this amino acid residue was only found in activated FiCAR1 cells. Moreover, another phospho S685 which is crucial for protein-protein interaction in T cell signaling was solely found in activated FiCAR1 (Mayya et al., 2009). Our findings are consistent with an interpretation that in FiCAR1 cells the high serine kinase activity of LCK initiates the activation of CD3ζ, consequently resulting in downstream phosphorylation of ZAP70 and as a result in phosphorylation of SLP-76 and phosphorylation of PLCγ1, and consequently activation of PKCθ leading to NFκB activation (Figure 6). The importance and involvement of these proteins in TCR signaling has been documented previously (Gomez-Rodriguez et al., 2014; Klammt et al., 2015; Bhattacharyya and Feng, 2020). It has been reported earlier that the TCR signal is strengthened by coupling it with the CD28 receptor (Xia et al., 2018). The FiCAR1 intracellular signaling domain also contains CD28. Figure 6 shows the phosphorylation of PDK1 and AKT which leads to the activation of AP-1. Although both PDK1 and AKT were found in activated mock and FiCAR1 reporter cells, threonine T) 245 in PDK and S122,124 and S137 phosphorylation in AKT only observed in FiCAR1 (Figure 7). These phosphorylation sites are important for mitotic phosphorylation (Dephoure et al., 2008) and cancer cell phospho-proteome identification (Zhou et al., 2013a). Phospho S241 in PDK is important for the activity of PI3K identification *in vivo* (Casamayor et al., 1999). It has also been proposed that the coupling of CD28 activates many downstream signaling pathways like the recruitment of phosphatidylinositol-3-kinase (PI3K) and AKT kinases which facilitates prolonged nuclear localization of NFAT, and thus IL-2 production and further phosphorylation of PLCγ (Shah et al., 2021). Again, phosphorylations were detected in the transcription factor NFAT factor both in mock and activated FiCAR1 samples, but no S or T phosphorylation was found in mock samples, they were only seen in FiCAR1 (Figure 7) samples meaning CAR engagement activates intracellular signaling which is crucial for T cell activation. Ras GTPases are involved in the activation of NFAT and translocation of NFAT to the nucleus (Woodrow et al., 1993) and activation of RasGTPase, regulates diverse cellular processes in T cells *via* linking to several biochemical effectors signaling pathways such as the Raf/MEK/ERK (Shah et al., 2021). VAV1 is a guanine nucleotide exchange factor (GEF) for small GTPases, such as Rac1, Rac2, and Rho, where it plays a crucial role in amplifying CD28-mediated activation of NFAT and NFκβ signaling pathways which is needed for prolonged T cell activation (Shah et al., 2021). VAV1 and Erk phosphorylation which is important for T cell activation and regulation only observed from activated FiCAR1 samples.

MAPK pathway generated by KEGG similarly shows a significant enrichment of phosphoprotein in activated FiCAR1 samples (Supplementary Figure S4) compared to mock samples. Our observations indicate that in FiCAR1 equipped Jurkat reporter cells, the signaling pathways activated *via* CD3ζz/CD28 include CD28-PI3K-AKT-PKCθ- NFκB and AP-1, LCK--SLP-76-GEF-PLCγ1-NFAT and as well *via* MAPK pathways. Certain new phospho-sites were identified only in activated FiCAR1 carrying cells which may play an important role and that should be analyzed in future studies for a better understanding of CAR function.

In summary, this study shows that the length of the novel FiCAR backbone can be modified by engineering it with one to three SIRPα derived IgG like domains, and the backbone is also functional when armed with scFvs targeting the HER-2 and GD2 antigens. We surmise that the malleable FiCAR backbone will be useful for targeting e.g. such cancer antigens where particular physical dimensions of CARs are advantageous. We also found Jurkat reporter cells to be a good tool for assessing issues in CAR expression, and tonic signaling, improving the efficacy of target cell killing and investigating CAR signaling mechanisms. We expect that the Jurkat reporter cells can be used to expedite the discovery and analysis of novel CAR.

## Data Availability

The original contributions presented in the study are included in the article/Supplementary Material, further inquiries can be directed to the corresponding author.
