# Supplementary material for: Using the Jurkat reporter T cell line for evaluating the functionality of novel chimeric antigen receptors
Source: Front Mol Med. 2023 Feb 22;3:1070384. doi: 10.3389/fmmed.2023.1070384 (PMC11285682; doi:10.3389/fmmed.2023.1070384)
Supplement: Supplementary file 2 [file Table1.pdf]

Supplementary Material

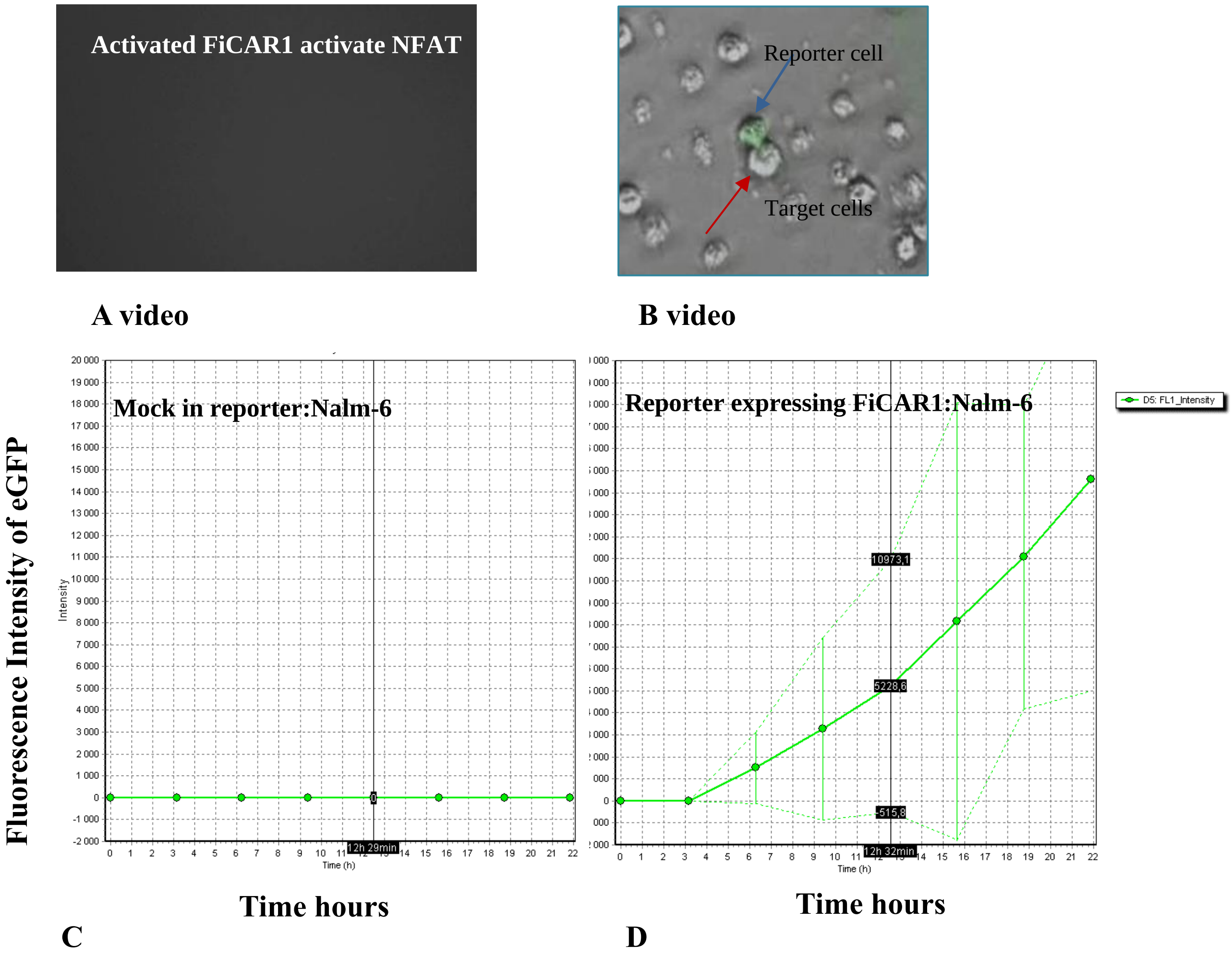

Supplemental Figure S1: Live cell microscopy of FiCAR1 expressed Jurkat reporter cells with Nalm-6 B-cells. Mock transduced Jurkat reporter cells were used as a negative control. **A.** Live cell imaging (video) by Cell-IQ high throughput microscope. **B.** Video of a FiCAR1 Jurkat cell killing aNalm-6 cell. **C, D.** Fluorescence intensity of eGFP was measured automatically as a function of time with the Cell-IQ analyzer software. The graphs show the increase in the fluorescence intensity of eGFP from mock and activated FiCAR1. The emergence of the eGFP signal indicates the activation of NFAT.

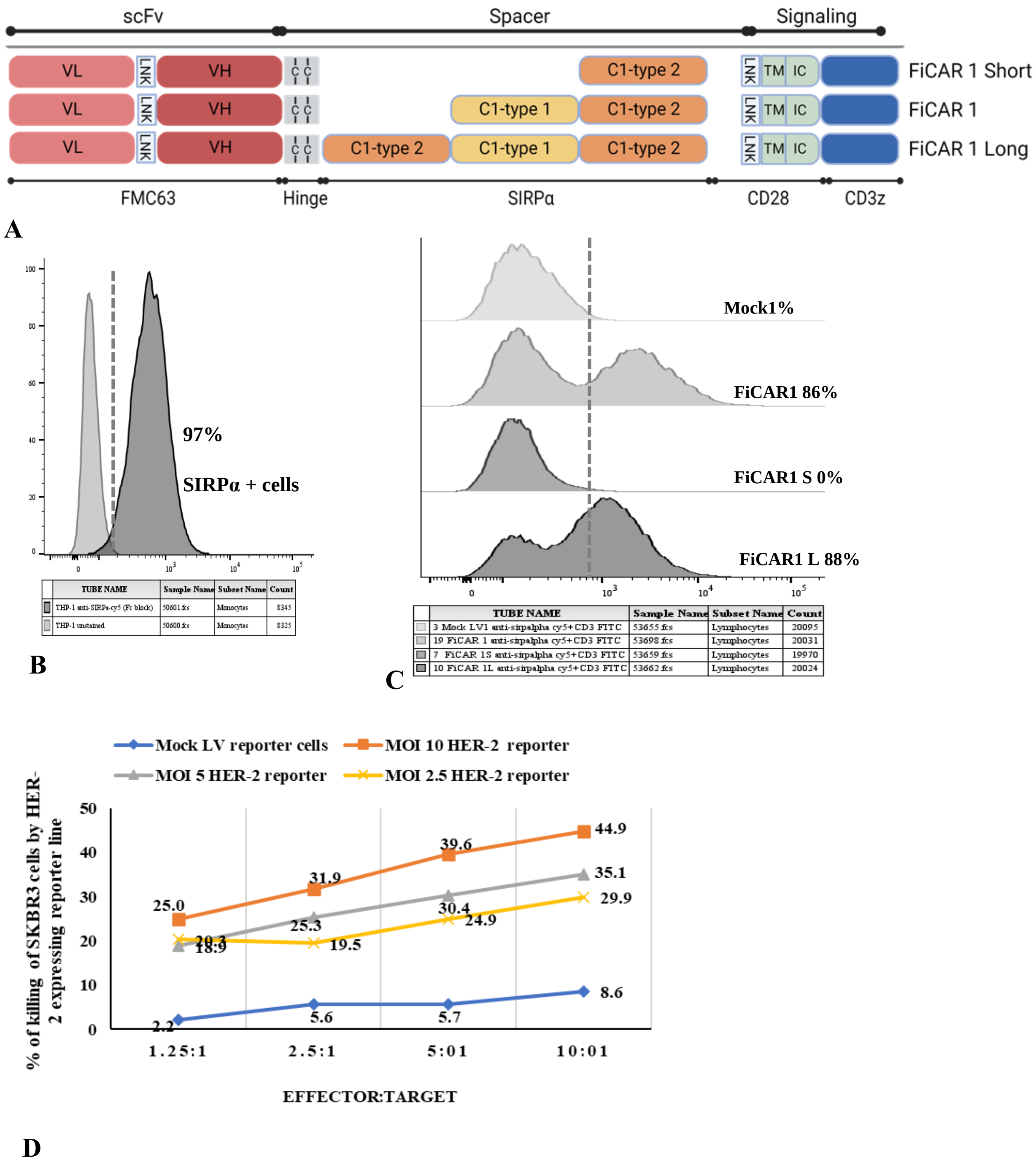

Supplemental Figure S2: Binding of SIRPα-Cy5 ab to different FiCAR versions. SIRPα (clone SE12B6A4) ab was used for the detection of FiCAR. **A.** Graphic structures of different versions of FiCARs were used in the experiment. **B, C** THP-1 **(B)** and mock and different FiCAR reporter cells **(C)** were stained with SIRPα ab and analyzed by flow cytometry. **(D)** Cytotoxicity against HER-2 positive SKBR3 cells of Jurkat reporter cells expressing HER-2 targeting FiCARs. Different MOIs of LV carrying the HER-2 gene have been used for the transduction and reporter cells expressing HER-2 were used as effector cells in this experiment. A reporter cell carrying mock LV was used as a negative control. The effector cells were co-cultured with luciferase-positive SKBR3 cells at various E:T ratios for 24 hours and the remaining live target cells were quantified by measuring luminescence.

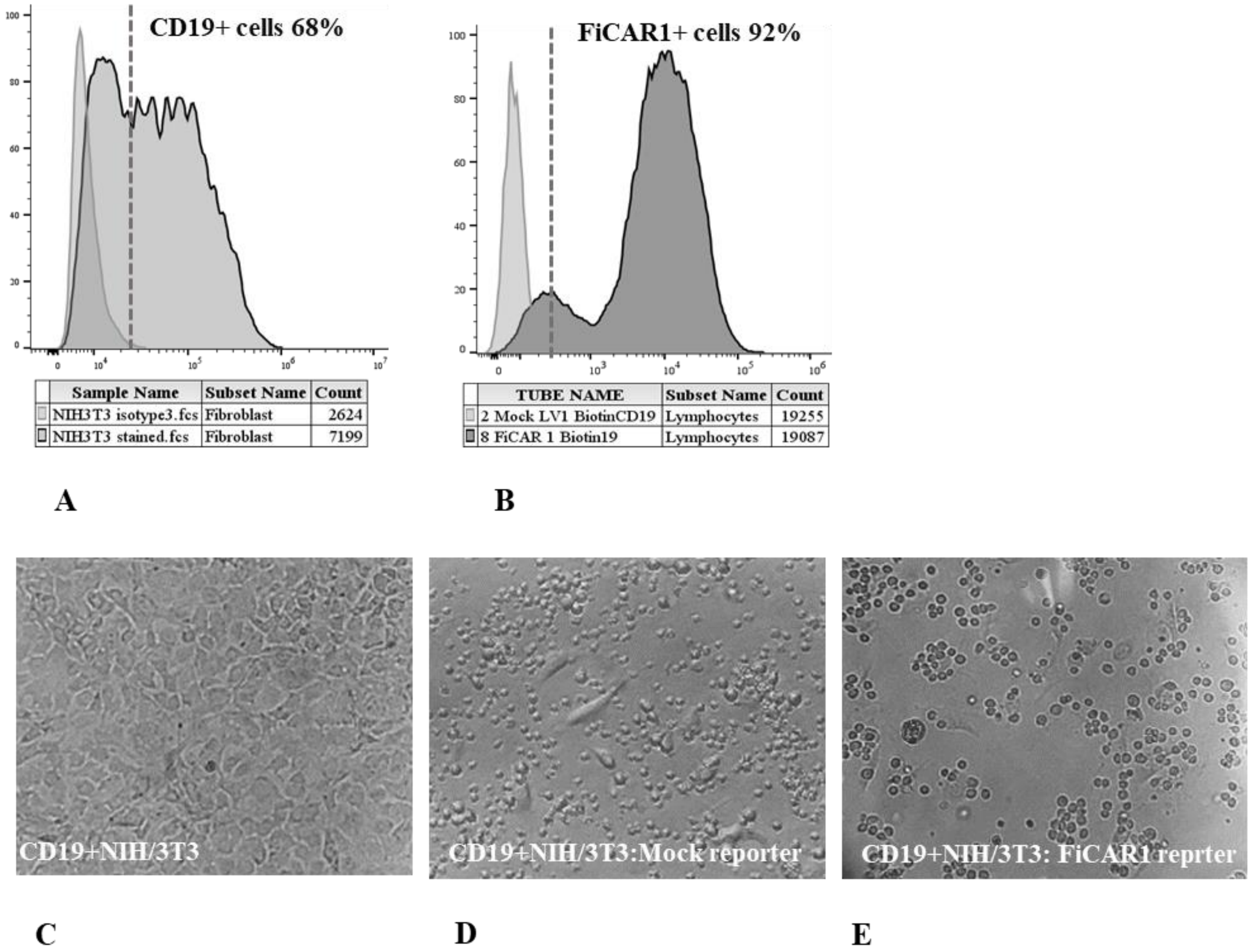

Supplemental Figure S3: Expression of CD19 in NIH/3T3 and FiCAR1 in Jurkat reporter cells for MS experiment. A, B. Cells were stained with CD19 ab and with CAR detection reagent and assessed with a DxFLEX flow cytometer. **C.** morphology of CD19+NIH/3T3 cells. **D, E.** cocultures of mock and FiCAR1 Jurkat reporter cells with CD19+ NIH/3T3 cells. Pictures were taken with an Olympus CKX53 microscope at 20X magnification.

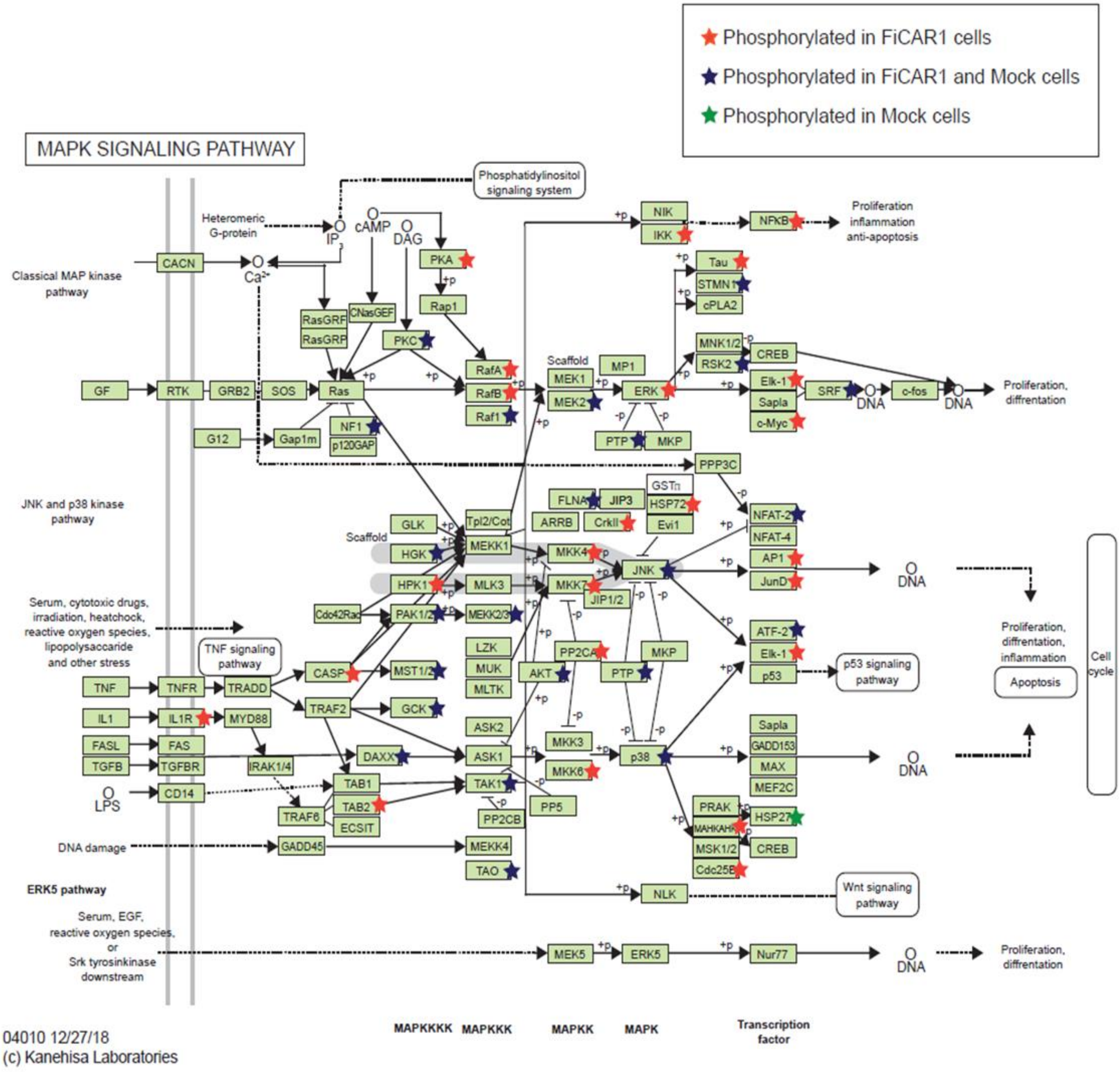

Supplemental Figure S4: The mitogen activated protein kinase (MAPK) pathway (information generated by KEGG, DAVID tool). Phosphorylation of the proteins in this pathway is indicated with stars. Red stars indicate phosphorylated proteins that were found in activated FiCAR1 samples; green stars indicate phosphorylated proteins that were found in mock samples, and blue stars indicate that the phosphorylated protein was detected in both conditions.
