# Supplementary material for: Using the Jurkat reporter T cell line for evaluating the functionality of novel chimeric antigen receptors
Source: Front Mol Med. 2023 Feb 22;3:1070384. doi: 10.3389/fmmed.2023.1070384 (PMC11285682; doi:10.3389/fmmed.2023.1070384)
Supplement: Supplementary file 3 [file Table1.DOCX]

Supplementary Material

**Supplemental Figure S1: Live cell microscopy of FiCAR1 expressed Jurkat reporter cells with Nalm-6 B-cells.** Mock transduced Jurkat reporter cells were used as a negative control. **A.** Live cell imaging (video) by Cell-IQ high throughput microscope. **B.** Video of a FiCAR1 Jurkat cell killing aNalm-6 cell. **C, D.** Fluorescence intensity of eGFP was measured automatically as function of time with the Cell-IQ analyzer software. The graphs show the increase of the fluorescence intensity of eGFP from mock and activated FiCAR1. Emergence of the eGFP signal, indicating activation of NFAT.

**Supplemental Figure S2: Binding of SIRPα-Cy5 ab to different FiCAR versions.** SIRPα (clone SE12B6A4) ab was used for the detection of FiCAR. **A.** Graphic structures of different versions of FiCARs used in the experiment. **B, C** THP-1 **(B)** and mock and different FiCAR reporter cells **(C)** were stained with SIRPα ab and analyzed by flow cytometry. **(D)** Cytotoxicity against HER-2 positive SKBR3 cells of Jurkat reporter cells expressing HER-2 targeting FiCARs. Different MOIs of LV carrying HER-2 gene have been used for the transduction and reporter cells expressing HER-2 were used as an effector cell in this experiment. Reporter cells carrying mock LV was used as a negative control. The effector cells were co-cultured with luciferase positive SKBR3 cells at various E:T ratios for 24 hours and the remaining live target cells were quantified by measuring luminescence.

**Supplemental Figure S3: Expression of CD19 in NIH/3T3 and FiCAR1 in Jurkat reporter cells** **for MS experiment. A, B.** Cells were stained with CD19 ab and with CAR detection reagent and assessed with a DxFLEX flow cytometer. **C.** morphology of CD19+NIH/3T3 cells. **D, E.** cocultures of mock and FiCAR1 Jurkat reporter cells with CD19+ NIH/3T3 cells. Pictures were taken with an Olympus CKX53 microscope at 20X magnification.

**Supplemental Figure S4: The mitogen activated protein kinase (MAPK) pathway (information generated by KEGG, DAVID tool).** Phosphorylation of the proteins in this pathway are indicated with stars. Red stars indicate phosphorylated proteins that were found in activated FiCAR1 samples; green stars indicate phosphorylated proteins that were found in mock samples, and blue stars indicate that the phosphorylated protein was detected in both conditions.
