# Supplementary figures and images for: Using the Jurkat reporter T cell line for evaluating the functionality of novel chimeric antigen receptors
Source: Front Mol Med. 2023 Feb 22;3:1070384. doi: 10.3389/fmmed.2023.1070384 (PMC11285682; doi:10.3389/fmmed.2023.1070384)

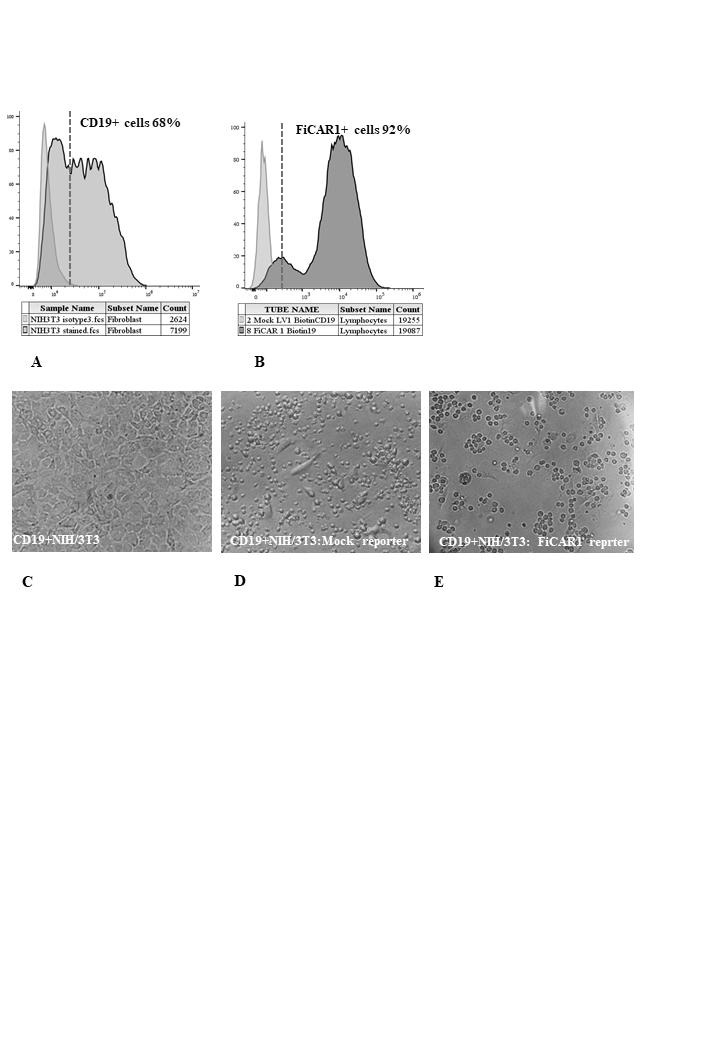

Supplement: Supplementary file 5 [file Image3.TIF]

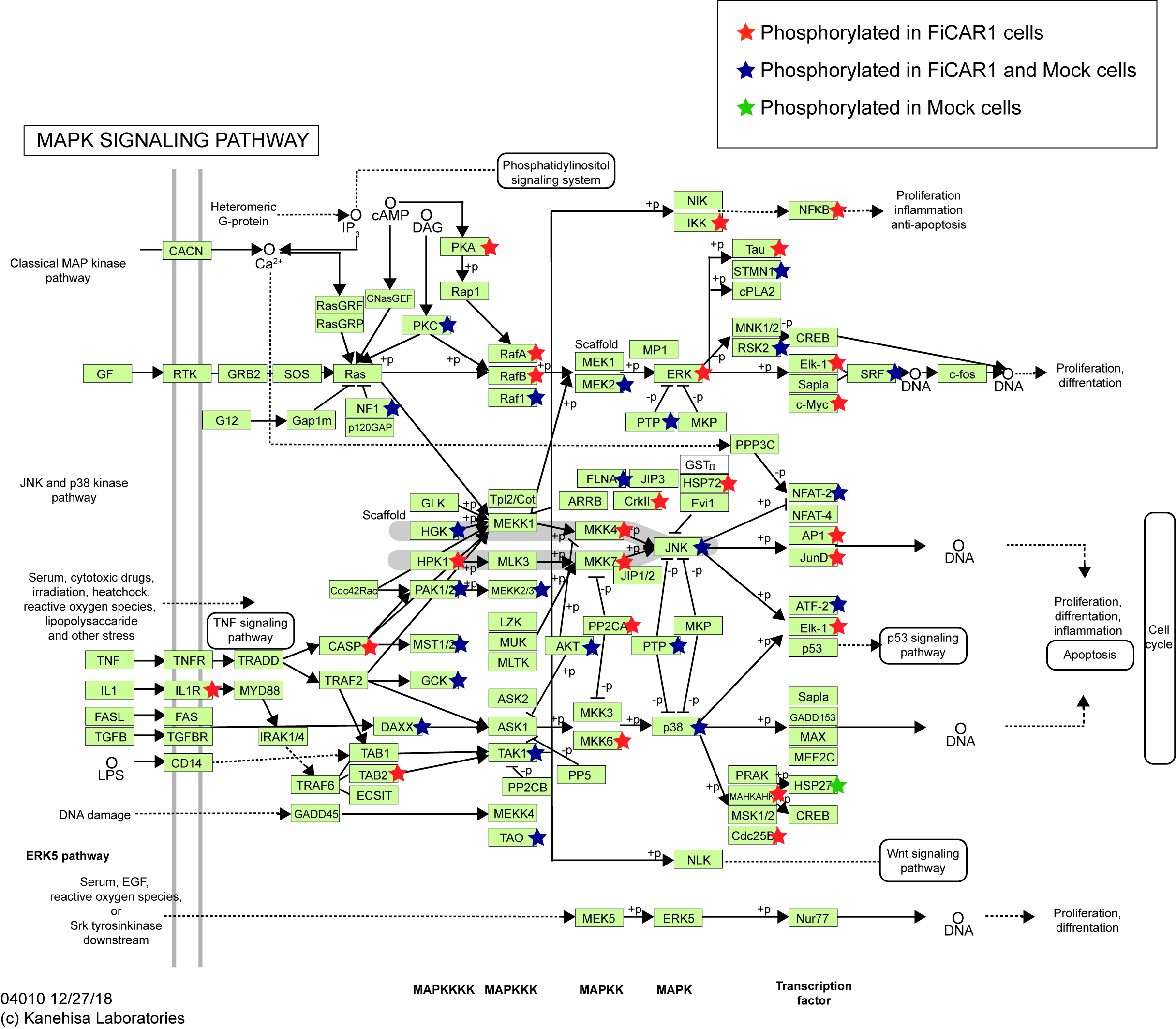

Supplement: Supplementary file 6 [file Image4.TIF]

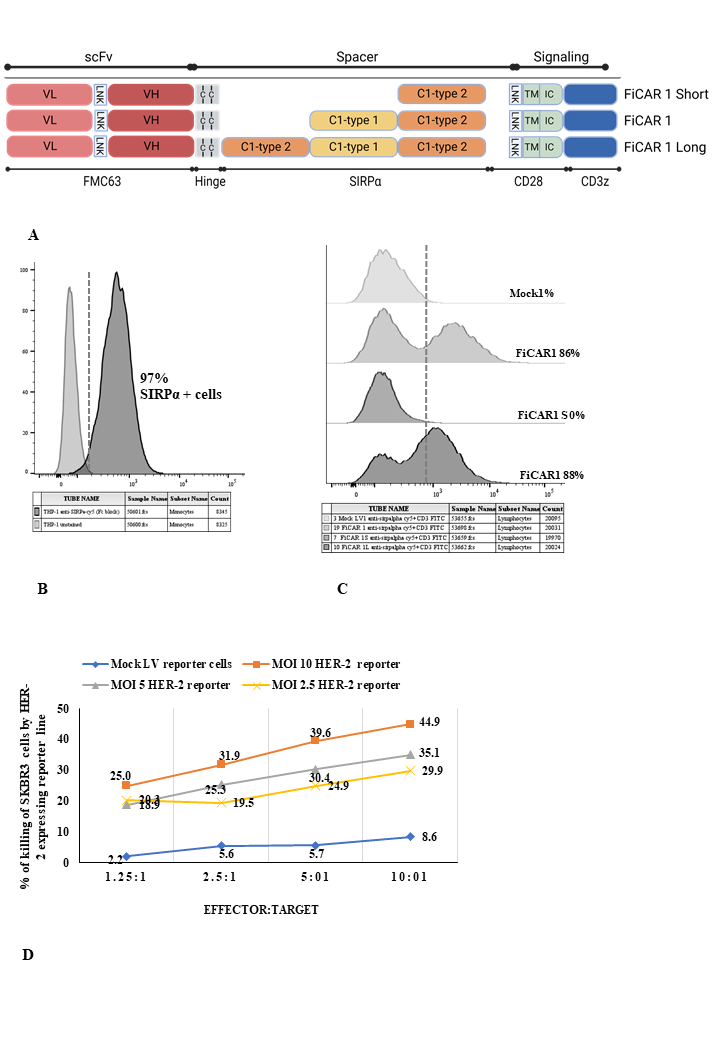

Supplement: Supplementary file 7 [file Image2.TIF]

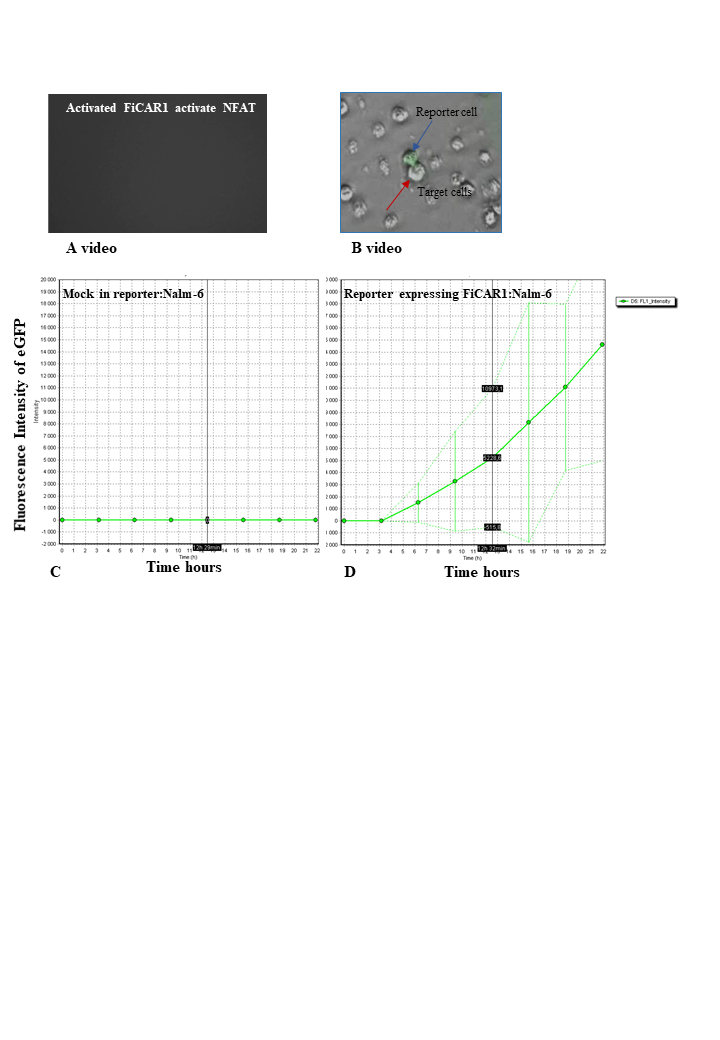

Supplement: Supplementary file 8 [file Image1.TIF]
